# Long term tillage regime alters bacterial assimilation of xylose and cellulose

**DOI:** 10.1128/aem.00933-25

**Published:** 2025-08-06

**Authors:** Marie Schaedel, Chantal Koechli, Daniel H. Buckley

**Affiliations:** 1 Soil and Crop Sciences, Department of Integrative Plant Science, Cornell University 251785 , Ithaca, New York, USA; Georgia Institute of Technology, Atlanta, Georgia, USA

**Keywords:** stable isotope probing, soil bacteria, carbon, tillage, life history

## Abstract

Microbial growth dynamics determine carbon fate in soil by transforming carbon inputs into microbial products available for stabilization on soil surfaces. Management practices such as tillage disturb microbial communities and promote C loss, but the degree to which tillage alters bacterial metabolism of soil C remains poorly described. We conducted a multi-substrate DNA stable isotope probing experiment using soil from a long-term field experiment with a 42-year legacy of either no-till or annual moldboard plowing. We predicted that this land use history would alter C assimilation dynamics due to differences in bacterial growth responses. We incubated soil from each tillage regime with ^13^C-xylose and ^13^C-cellulose, substrates that differ in bioavailability and which favor different bacterial life history strategies in soil. We identified 730 ^13^C-labeled bacterial taxa and tracked their abundance in bulk soil over a 30 day period. Carbon addition to soil rapidly altered bacterial community structure and function. ^13^C-labeling dynamics differed substantially between tilled and no-till soils with respect to both xylose and cellulose. Bacterial xylose metabolism in tilled soils exhibited substantial lag relative to no-till soils, and this lag corresponded with lower mineralization rates for xylose. In addition, bacterial cellulose metabolism was mediated primarily by specialist taxa in no-till soils, while dual incorporators dominated tilled soils. Differences in carbon assimilation corresponded to lower cellulose mineralization rates and cumulative cellulose mineralization in tilled soils. We show that soil management practices shape the path of carbon through bacterial communities by altering dynamic growth responses associated with bacterial life history strategies.

**IMPORTANCE:**

We applied DNA stable isotope probing in a microcosm experiment to understand the role of soil management (till vs no-till) in shaping bacterial carbon cycling. Our hypothesis was that a legacy of disturbance through tillage would exert a selective influence on bacterial growth dynamics, thereby altering bacterial processing of added carbon substrates. We found that lagged growth in tilled soil resulted in delayed bacterial assimilation of xylose and a streamlined, single carbon “channel” characterized by the co-metabolism of xylose and cellulose. In no-till soil, temporally distinct bacterial assimilation of xylose and cellulose by separate carbon “channels” was associated with higher carbon mineralization rates and total mineralization relative to tilled soil. Our findings indicate that soil management practices altered the growth dynamics of active carbon cycling bacteria. Lagged growth associated with a history of disturbance resulted in reduced carbon mineralization.

## INTRODUCTION

Agriculture is a significant global source of greenhouse gas emissions, and the ongoing conversion of natural landscapes to agriculture has outsized impacts on terrestrial losses of soil organic carbon (SOC) (1). Agricultural landscapes represent nearly 40% of global terrestrial area (2), and it is estimated that agricultural activities to date have released as much as 90 pg carbon into the atmosphere (3). Soil microbial communities (bacteria and fungi) act as arbiters in determining the fate of carbon (C), with microbial growth response mediating the transformation of C inputs (4). Living microbial communities facilitate the breakdown of C-containing plant litter into altered biochemical forms (5). Such processes are fundamental to the decomposition of plant litter and its transformation into SOC (6) and stable forms of organic matter (7). Therefore, improving our understanding of how agricultural practices impact microbial C cycling is necessary to address current trends in SOC loss.

Conservation agriculture practices such as no-till have been associated with SOC sequestration (8, 9) and have documented belowground impacts on soil microbial communities (10, 11). Soil microbial diversity has been associated with improved carbon use efficiency, implying a functional connection between community structure and C cycling (12). Although differences in microbial community structure between plow-till and no-till soils are thought to account for differences in C cycling, direct evidence is lacking (13–15) due to the inability of DNA sequencing methods to resolve *in situ* activity. Furthermore, the C sequestration potential of no-till practices has been increasingly called into question (16, 17) as microbial community responses to such practices tend to be context dependent. Improving our mechanistic understanding of how agricultural management alters microbial C cycling could allow us to better manage for SOC accumulation via microbial pathways.

Stable forms of carbon that have soil residence times measured in decades largely derive from “dead” microbes (18). If microbial necromass comprises a large proportion of SOC, then microbial growth dynamics are a major determinant of SOC persistence. Microbial growth-death cycles and community turnover are, therefore, fundamental to the accumulation of SOC and its cycling in soil (19, 20). However, the relationship between microbial growth dynamics and the pathways by which microbial communities transform C in agricultural soils remains unclear. Understanding how soil management practices alter bacterial growth responses to carbon inputs could improve our ability to manage soils for improved SOC stabilization potential.

16S rRNA copy number (*rrn*) has been used as a predictor of growth dynamics and C use efficiency in bacteria (21, 22). Growth-adapted, or ruderal, bacteria contain multiple *rrn* copies in their genomes to rapidly achieve high ribosomal content, enabling them to respond rapidly to transient increases in resource availability (21, 23). Bacteria with different *rrn* copy numbers exhibit distinct growth responses to C substrates with varying levels of bioavailability (24, 25). For instance, ruderal bacteria with high *rrn* grow rapidly to mineralize bioavailable sugars (glucose, xylose), while slower-growing organisms with lower *rrn* tend to mineralize insoluble and low bioavailability substrates (cellulose, vanillin) gradually without rapid variation in population size (24). While inferred *rrn* from complex environmental samples should be interpreted cautiously, these data have proved useful in generating new ecological insights into the functional significance of growth-adapted taxa across ecological contexts (26, 27).

Although microbial growth and death cycles are deterministic of the fate of C in terrestrial landscapes, we have only recently begun to elucidate linkages between land-use, microbial growth dynamics, and C use strategies. Agricultural systems are often characterized by high disturbance management practices such as tillage that alter SOC stocks and exert selective pressure on C-cycling microorganisms (11, 27). Tillage mechanically disrupts soil aggregates and accelerates C loss by promoting its rapid oxidation by aerobic microorganisms (28, 29). Compared with undisturbed sites, bacterial communities in agricultural fields experience significant declines in diversity in response to C addition, driven principally by the proliferation of *Gammaproteobacteria* (30). Life history frameworks predict that disturbance disfavors taxa that compete on the basis of resource acquisition (31). Hence, we would expect disturbed soils to disfavor taxa that assimilate C sources of lower bioavailability such as cellulose. Indeed, DNA stable isotope probing (SIP) has revealed that bacteria in agricultural fields preferentially assimilate bioavailable forms of C such as xylose over insoluble forms such as cellulose (30). Accumulating evidence, thus, implies that land-use alters bacterial growth dynamics and C cycling.

We conducted a microcosm experiment using ^13^C-xylose and ^13^C-cellulose addition to soils from a long-term field experiment with a 42-year management history of continuous corn cropping with or without moldboard plowing. Both sets of fields are managed with biomass removal, resulting in no-till biomass harvested (NTH) and plow-till biomass harvested (PTH) treatments. Past research at this long-term experimental site has demonstrated significant tillage effects on SOC and organic nitrogen pools (32). Xylose and cellulose were selected as substrates for this experiment because they are primary components of plant cell walls which differ in their bioavailability, primarily due to differences in solubility. The bioavailability of C substrates is closely related to bacterial growth dynamics, with ruderal organisms rapidly metabolizing soluble forms of C and slower-growing competitor species being more active in mineralizing insoluble C (24).

We hypothesized that a long-term tillage regime would exert selective pressure on microbial communities due to disturbance (31). Specifically, we hypothesized that disturbance would alter C mineralization and assimilation dynamics by favoring growth-adapted ruderal taxa that preferentially assimilate soluble substrates such as xylose and disfavoring taxa that preferentially assimilate insoluble substrates such as cellulose. We predicted that these effects would be evidenced by higher xylose mineralization in plow-tilled soils relative to no-till, concurrent with a higher abundance of xylose-assimilating taxa. Similarly, we expected to observe greater cellulose mineralization in no-till soils, concurrent with a higher abundance of cellulose-assimilating taxa.

## MATERIALS AND METHODS

### Soil sampling

We sampled soils from the long-term tillage research plot at the Miner Institute in Chazy, NY (Clinton County, 44°53.13′N, 73°28.40′W). Plots (6 × 15.2 m) were established in 1973 and arranged in a randomized complete block design consisting of four replicates, managed identically with the exception of imposed tillage regime. All treatments were planted to maize and fertilized according to standard agronomic practices in the region (26 kg Ha^−1^ N per year). Harvest occurred in mid-October and tillage occurred by mid-November; plant biomass was removed for silage by cutting stalks adjacent to the soil surface with roots remaining in place. Soil characteristics of NTH and PTH field soils are provided in Table S1.

Soils for stable isotope probing were sampled in September 2014 from no-till, biomass harvested (NTH) and plow-till, biomass harvested (PTH) plots to isolate the effects of tillage. We collected 20 topsoil cores (5 cm depth) across each replicate plot. Cores from a unique replicate plot were combined and homogenized through sieving (2 mm sieve). Soil was placed on ice during transport and stored at 4°C until the beginning of the experiment (3 days). We also collected soil moisture and temperature data for three replicates within each plot.

### Microcosm set-up and experimental design

Microcosms were set up in 250 mL Erlenmeyer flasks. Ten grams of dry weight soil were placed in the flasks and sealed with butyl rubber stoppers to prevent moisture loss. Incubations were performed in the dark at room temperature. Dry weight was determined by gravimetric soil moisture measurements for three technical replicates within each tillage treatment and biological replicate (33). Microcosms were pre-incubated for 2 weeks until the production of CO_2_ stabilized following disruption due to sieving, as assessed by GC-MS analysis (Shimadzu QP2010S GC-MS plumbed with Carboxen-1010 PLOT column, St. Louis, MO). Carbon substrates for microcosm enrichment were chosen based on the composition of corn stover (34) due to the long-term cropping history of corn in the field site where soils were collected. Carbon substrates were added in a solution containing Murashige and Skoog basal salt mixture to 50% of the water holding capacity of the soil. The ^12^C control treatments contained carbon with a natural abundance of ^13^C. Water-only control microcosms were treated with an equivalent amount of water and basal salts as other treatments to serve as a control for moisture effects. Additional details can be found in Supplemental Methods.

**Fig 1:**
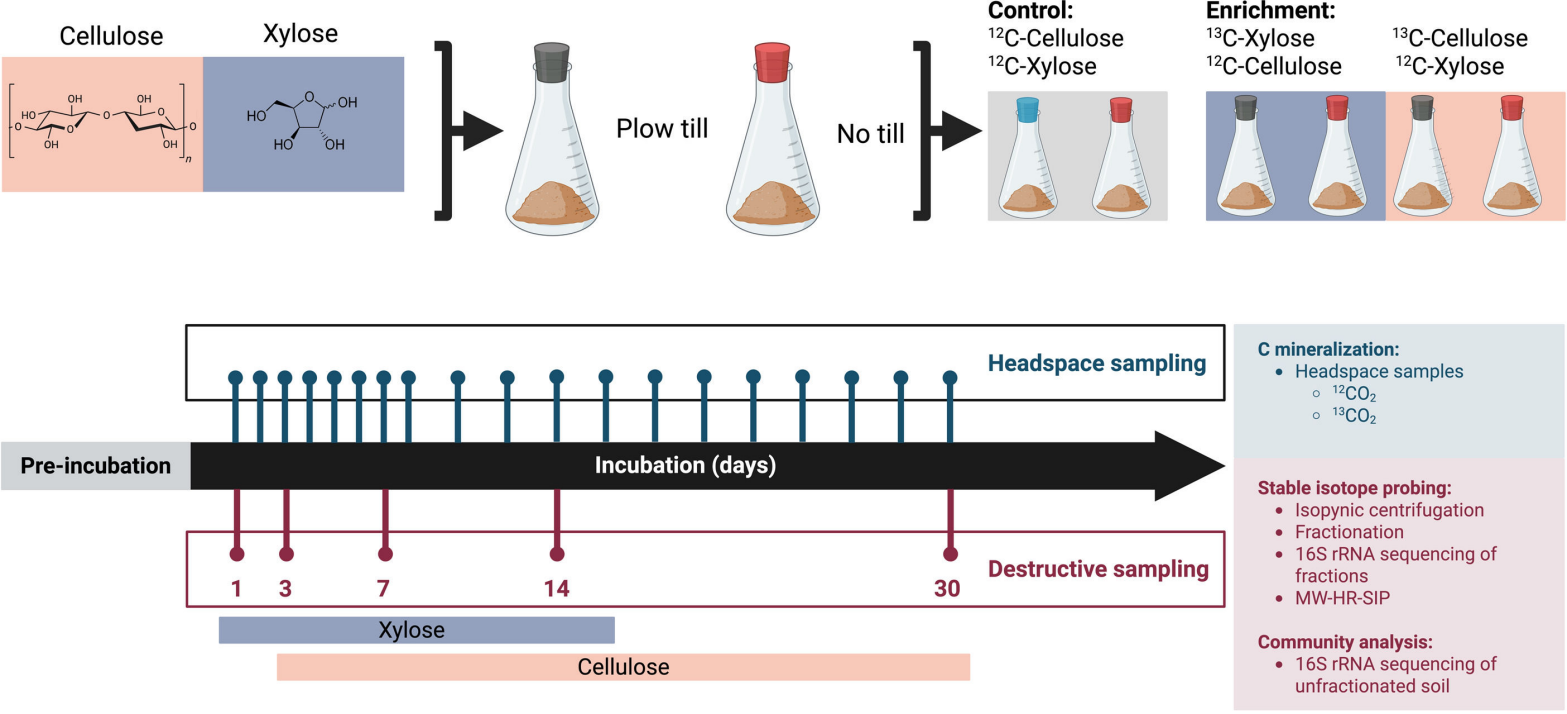
Schematic of microcosm experimental design. Soil was collected from contrasting tillage histories and used in DNA-SIP microcosms. All microcosms received the same set of carbon substrates (xylose and cellulose), but only one substrate was ^13^C-labeled in the enriched microcosms. Microbial community analysis was performed on all treatments and times for four field replicates (*n* = 112). Microcosm headspace sampling was used to determine mineralization activity of individual substrates and cumulative mineralization of all substrates. Destructive sampling of microcosm soil over time was used to assess changes in bacterial community structure and C assimilation dynamics.

Microcosms (*n* = 112) were prepared using soil derived from field replicates (*n* = 4). Unlabeled control microcosms and water only controls were destructively sampled five times at 1, 3, 7, 14, and 30-days following substrate addition. Microcosms receiving ^13^C cellulose were sampled on days 3, 7, 14, and 30, while ^13^C xylose microcosms were sampled on days 1, 3, 7, and 14 (Fig. 1). We limited our destructive sampling times to expected windows of substrate mineralization activity for xylose and cellulose. These sampling timepoints were selected because results from this and previous studies (24, 25, 30, 35) indicate that xylose is completely mineralized by day 14 while appreciable cellulose mineralization occurs only after 3 days. A total of eight water-only microcosms were prepared as controls with four replicates from each tillage regime. Water-only controls were used to monitor baseline mineralization in the absence of carbon and were destructively sampled at the end of the experiment on day 30. Soil from harvested microcosms was stored at –80°C until DNA extraction was performed.

### Carbon mineralization

Stoppers were removed from microcosms every 2 days, and the headspace was flushed with filtered (0.2 µm) air. ^12^CO_2_ and ^13^CO_2_ efflux resulting from soil microbial respiration was measured non-destructively at multiple timepoints throughout the 30-day incubation (Fig. 1). Two milliliter gas sampling vials flushed with helium were used to collect 0.25 mL of microcosm headspace. Headspace samples were analyzed on a Shimadzu GP2010S GC-MS (St Louis, MO) with an injection port temperature of 200°C. The quadrupole MS was run in selective ion mode (SIM), scanning for *m*/*z* of 44 (^12^CO_2_), 45 (^13^CO_2_), and total ion count (TIC). A CO_2_ standard curve was prepared for each sample batch and was used to quantify CO_2_ content in the microcosm headspace. ^13^CO_2_ measurements were used to monitor mineralization activity of individual labeled substrates, while ^12^CO_2_ and ^13^CO_2_ were used to monitor total mineralization activity of all added carbon substrates.

### DNA extraction

We extracted DNA from bulk (unfractionated) microcosms using 2 × 0.25 g of soil from all replicates of each isotope × day × soil combination (112 samples). We used a modified Griffiths phenol-chloroform extraction procedure (36), as detailed in Supplemental Methods. Following extraction, DNA extracts were cleaned using illustraMicroSpin G-50 columns (GE Healthcare; Buckinghamshire, UK; 27-5330-02) and magnetic bead purification (Agencourt AMPure XP purification; Beckman Coulter; Brea, CA; A63880) according to manufacturer protocols. DNA for isopycnic centrifugation was extracted from four technical replicates of 0.25 g of soil, following the phenol-chloroform procedure outlined above. Technical replicates of DNA extractions were pooled and selected for a size of 4–14 kb with a Blue Pippen Prep machine (Sage Science, Beverly, MA) to promote uniform density equilibration according to DNA molecular weight (37, 38).

### Stable isotope probing and isopycnic centrifugation

DNA-SIP was performed for a total of 42 samples. Most DNA-SIP studies are performed at a single time point, which is problematic because microbial responses to carbon inputs are highly dynamic. Unfortunately, DNA-SIP is a labor-intensive method which does not lend itself to heavy replication across time. Hence, we chose to perform DNA-SIP across time in a single field replicate, while performing field level replication of DNA-SIP at critical time points. Hence, both treatments and all isotope by time combinations were determined for one field replicate (as indicated in Fig. 1, *n* = 26), while three replicates were used to assess treatment level differences for ^13^C-xylose on day 3 and for ^13^C-cellulose on day 30. We prepared isopycnic gradients as described previously (24, 30). Size-selected DNA was centrifuged in a CsCl density gradient on an Optima MAX-E ultracentrifuge (Beckman Coulter; Brea, CA). Following centrifugation, 100 µL density fractions were collected and prepared for sequencing. Detailed methods are provided in Supplemental Methods.

### 16S rRNA amplicon sequencing

We performed amplicon sequencing of the V4 region of 16S rRNA gene across all unfractionated (*n* = 112) and fractionated (*n* = 979) samples. We amplified the V4 hypervariable region of 16S rRNA gene using dual-indexed primers (515f/806r) (39). The 16S rRNA libraries were prepared as described previously and in Supplemental Methods. Pooled amplicon libraries were submitted for sequencing at the Cornell Core Facility in Ithaca, NY. Samples were run on an Illumina MiSeq using V2 chemistry with 2 × 250 bp read length.

Raw sequence data were processed using QIIME2 v2023.9.1 (40). Fractionated samples from SIP and unfractionated microcosm samples were processed concurrently in the same pipeline to enable amplicon sequence variant (ASV) cross-referencing between SIP fractions and unfractionated microcosm samples. As described previously (27), sequences were demultiplexed and trimmed based on an examination of the quality scores. Quality filtering, denoising, and ASV merging were performed with DADA2 v2023.9.1. Phylogenetic assignment of ASVs was performed using the Silva v138 database (41). We used MAFFT 7 (42) to align sequences and FastTree 2 (43) to construct a phylogenetic tree.

Processed sequences were imported into R version 4.0.3 (44). ASV count data from unfractionated bulk microcosm samples were rarefied to an even depth of 1,567 in phyloseq version 1.34.0 (45) using the *rarefy_even_depth* function. Alpha diversity indices were calculated using *estimate_richness* in phyloseq. Pielou’s evenness was calculated as the ratio of Shannon diversity to the natural log of richness per sample. Weighted and unweighted Unifrac distances were calculated with the *Unifrac* function in phyloseq and Bray-Curtis distances were generated with *avgdist* in vegan (version 2.5.7) (46). The estimated 16S rRNA copy number (*rrn*) for each ASV was determined using paprica (47). Faith’s phylogenetic diversity was determined using *pd* from the picante package (48).

### Identification of ^13^C-labeled taxa

MW-HR-SIP was performed using the HTSSIP package (49) in R. ASV count data were pruned in phyloseq using the *filter_taxa* function prior to identifying incorporators to retain ASVs that occurred at least twice across all samples. Incorporators were identified using the R package HTSSIP (49), which includes wrapper functions from DESeq2 (50) to identify ASVs that are significantly enriched in “heavy” gradient fractions in ^13^C treatments relative to “heavy” fractions in ^12^C controls. Independent sparsity filtering was used to remove statistically uninformative taxa at a sparsity threshold that was determined independently for each tillage × timepoint × substrate comparison (49). Remaining taxa were deemed incorporators if they demonstrated a log_2_-fold enrichment of 0.25 in heavy fractions of treatment (^13^C) samples relative to corresponding fractions of control (^12^C) samples. Overlapping buoyant density windows of 1.70–1.73, 1.72–1.75, and 1.74–1.77 g mL^−1^ were used based on a prior analysis (51) that identified these windows as having the highest detection rate while minimizing false-positives. This approach has been validated (51) and described in detail elsewhere. We used Venn diagrams to visualize shared incorporators by tillage regime and substrate with the eulerr package in R (52). ^13^C incorporators were identified in microcosm soil communities according to their ASV identifier for subsequent analyses.

### Statistical analysis

#### Carbon mineralization

We evaluated the contribution of tillage history and days since C addition on cumulative mineralization (mg C) and mineralization rates (mg C h^−1^) of individual substrates (^13^C) and combined substrates (^12^C+^13^C) within the microcosm headspace. We used linear mixed effects models from the lme4 package (53) in R version 4.0.3 (44), specifying sample day, tillage regime, and their interaction as fixed effects and replicate as a random effect. The significance of fixed effects within the models was assessed with *anova* from the stats package (44). Contrasts between tillage regime were determined using *emmeans* from the emmeans package (54) and corrected *P*-values for multiple comparisons with the Benjamini-Hochberg adjustment. Post-hoc means separation was used to assess endpoint differences in cumulative ^13^C mineralized for each substrate and in total for all substrates (^12^C and ^13^C) by tillage regime. The chosen endpoints for each substrate were day 14 for xylose and day 30 for cellulose.

#### Microcosm community composition

We investigated the change in community composition following C addition in unfractionated microcosm samples by computing alpha and beta diversity at each timepoint. The change in diversity was computed as the difference between microcosms receiving a C substrate solution (^12^C + ^13^C) and the baseline water-only control that did not receive carbon. We employed linear mixed effects models as described previously to identify the contribution of tillage, sampling day, and their interaction in driving changes to diversity. Significance was determined using Benjamini-Hochberg corrected *P*-values of less than 0.05. Homogeneity of dispersion across tillage regimes was determined using *betadisper* and tested for significance with *permutest* from vegan. We identified differentially abundant taxa using a Maaslin2 (55) model in which tillage, sampling day, and their interaction was specified as fixed effects and replicate was specified as a random effect.

#### Growth responses and life history traits of incorporator taxa

Maximum log_2_-fold change (max l2fc) for incorporator ASVs was determined using normalized abundance values for each ASV in bulk microcosm soil. ASV relative abundance was divided first by predicted *rrn* and then by the estimated *rrn* of the entire sample, as described previously (24) (Supplemental Methods). These normalized abundances were weighted by DNA yield (ng µL^−1^), which was measured with the Quant-iT PicoGreen dsDNA Assay Kit (Thermo Fisher Scientific). While DNA yield is not a direct measure of bacterial abundance, we use it as a proxy for microbial biomass based on prior work demonstrating strong correlations with microbial cell numbers (56–58). Max l2fc was calculated for each ASV as the difference between baseline abundance in untreated control soil (receiving H_2_O and micronutrients but no carbon) and the maximum recorded abundance after C addition in bulk soil. If the maximum normalized abundance of an incorporator in treated soil was less than its baseline abundance in untreated soil, max l2fc for that incorporator was assigned a value of zero.

We defined latency for taxa as the difference in time between the day of peak mineralization for a given substrate and the first day of ^13^C-labeling for a given taxon. Peak mineralization for xylose was observed on day 2, while peak mineralization for cellulose was observed on day 6. ASVs that had a detected ^13^C label before the time of peak mineralization were assigned a latency value of zero, indicating that they assimilated ^13^C on or before the time of peak mineralization. The degree of ^13^C assimilation was assigned for each incorporator ASV as the log_2_-fold change in abundance between ^12^C and ^13^C SIP density fractions (59).

We used Wilcoxon rank-sum tests for 2-factor comparisons evaluating incorporator traits according to substrate and tillage (*wilcox.test* in the stats package; R Core Team, 2020). Wilcoxon tests were used to evaluate differences in predicted *rrn,* latency*,* species richness, and Faith’s phylogenetic diversity (calculated using *pd* from picante) among incorporators from divergent tillage regimes (48).

Lastly, we assessed relationships between C cycling dynamics and phylogeny using functional distance matrices for incorporator ASVs in PTH and NTH microcosms. Carbon substrate (xylose or cellulose), the day of label detection, and the degree of assimilation (log_2_-fold enrichment in heavy fractions) were considered functional responses relevant to C metabolism. These values were scaled using *decostand,* and functional distance was calculated using *vegdist(method = “altGower”*) in vegan. Phylogenetic distance between incorporator ASVs was determined using *cophenetic.phylo* in phyloseq (45). We evaluated the relationship between phylogenetic and functional distance among incorporator taxa for each tillage regime separately using with Spearman rank correlation.

## RESULTS

### Mineralization dynamics of xylose and cellulose

CO_2_ production (as ^12^C + ^13^C) was tracked over time following the addition of cellulose and xylose. Cumulative C mineralization rates differed with respect to tillage on days 1 and 2, with no-till (NTH) soils exhibiting higher total mineralization rates relative to plow-till (PTH) soils (Fig. S1B). C mineralization rates were influenced by tillage (mixed effects ANOVA, *P* < 0.001), time (mixed effects ANOVA, *P* < 0.0001), and their interaction (mixed effects ANOVA, *P* < 0.0001; Table S2). The total amount of mineralized C (mg ^12^C + ^13^C) differed with respect to tillage history (mixed effects ANOVA, *P* < 0.0001) and time (mixed effects ANOVA, *P* < 0.001; Table S3).

**Fig 2:**
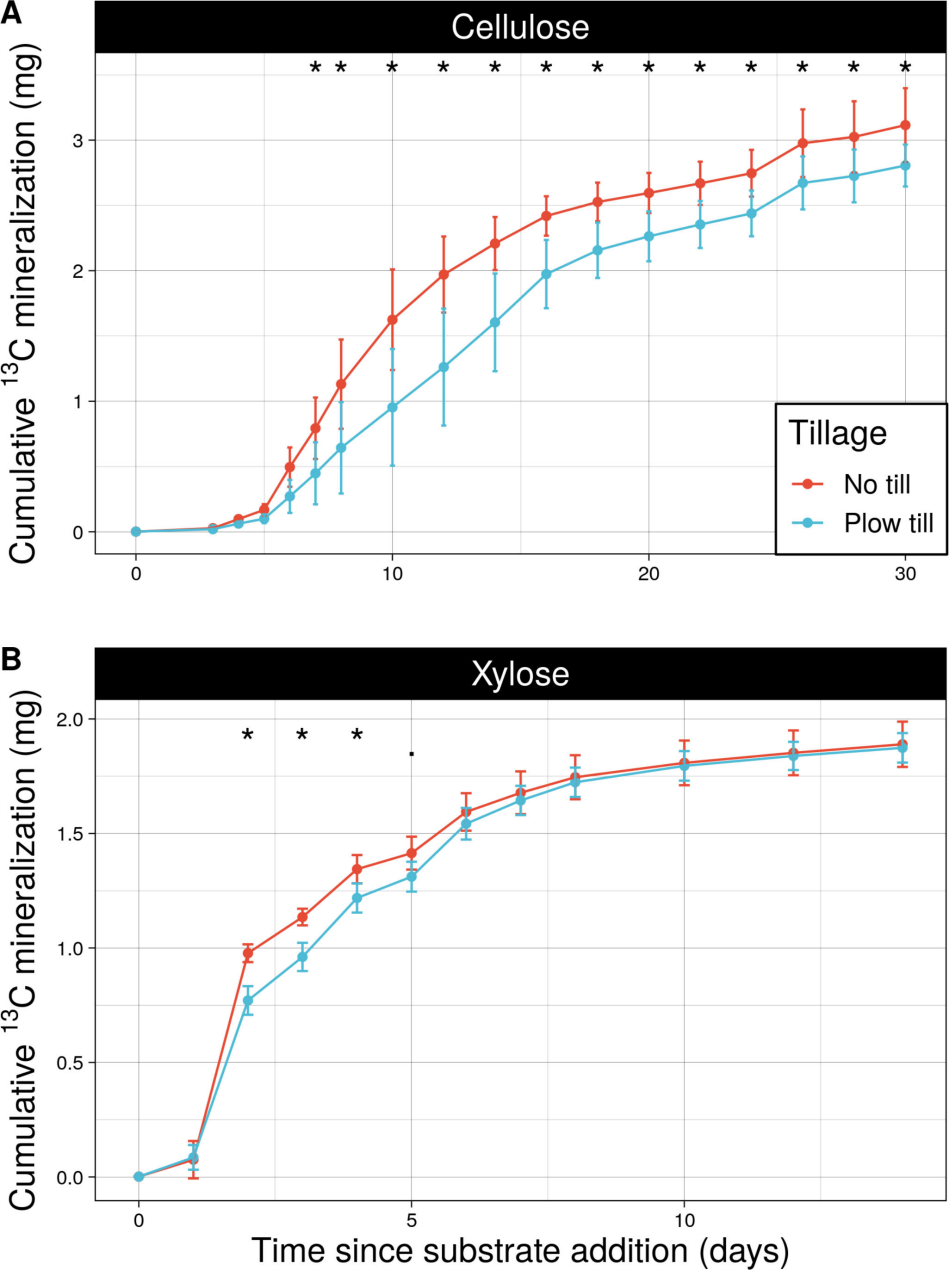
Cumulative amount of carbon (mg) mineralized for individual substrates of cellulose (**A**) and xylose (**B**) over time differed by tillage history. Asterisks (*) indicate significant differences by tillage history (adjusted *P*-value < 0.05) as determined by post-hoc pairwise comparisons of linear mixed effects models. Error bars denote standard deviation, while tillage regime is indicated by color.

**Fig 3:**
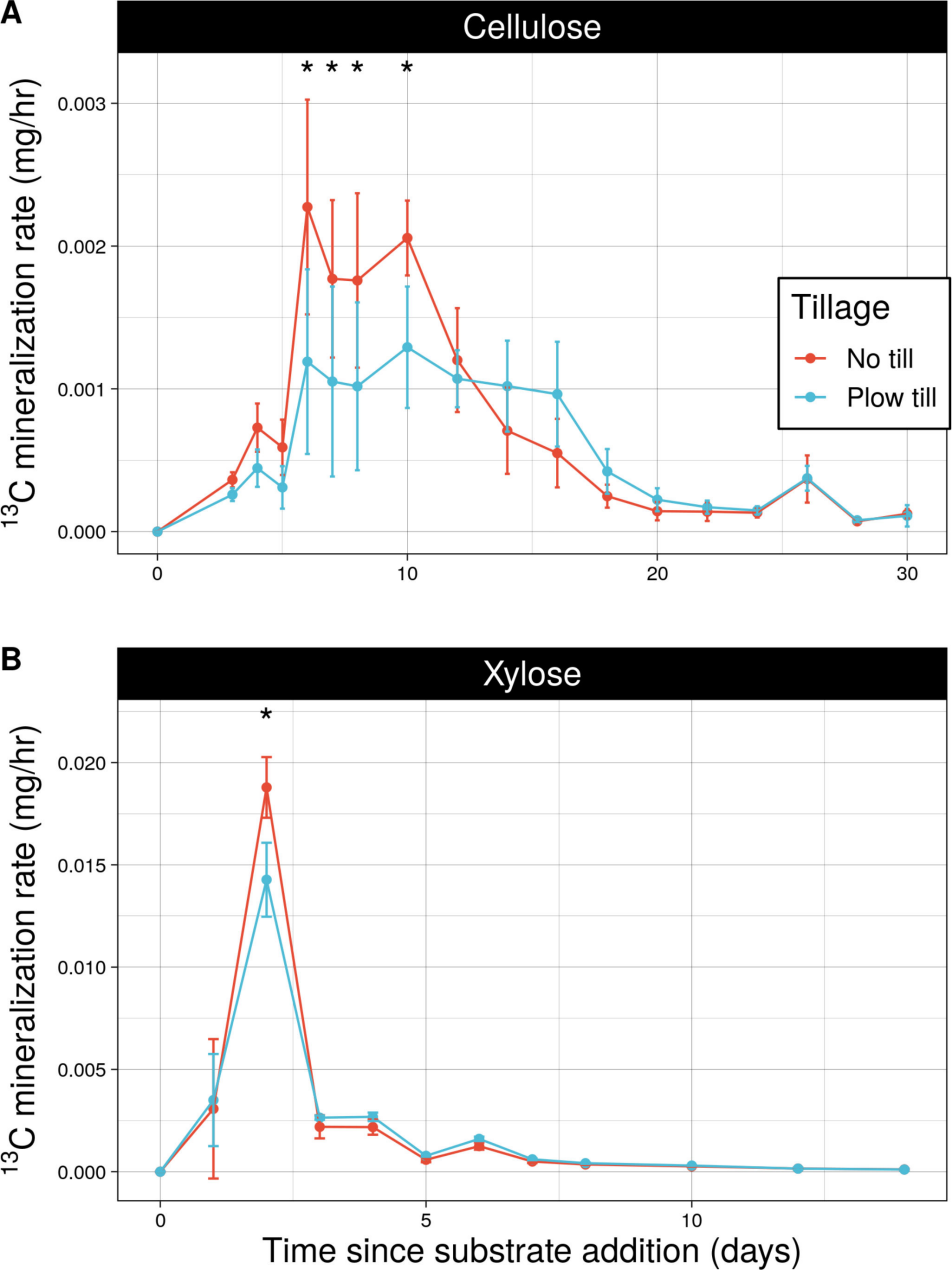
Carbon mineralization activity (mg h^−1^) of cellulose (**A**) and xylose (**B**) over time differed by tillage history. Asterisks (*) indicate significant differences by tillage history (adjusted *P*-value < 0.05) as determined by post-hoc pairwise comparisons of linear mixed effects models. Error bars denote standard deviation, while tillage regime is indicated by color.

Tillage regimes differed in cumulative ^13^C-cellulose mineralized from day 7 through day 30 following substrate addition (Fig. 2A), driven by NTH having significantly higher cellulose mineralization rates than PTH between days 6 and 10 (Fig. 3A). Tillage regimes also differed in cumulative mineralized ^13^C-xylose (Fig. 2B), driven by NTH having significantly higher xylose mineralization rates than PTH on day 2 (Fig. 3B). By the sampling endpoints (day 14 for xylose, day 30 for cellulose), NTH communities mineralized more ^13^C-cellulose than PTH communities (Tukey test, *P* < 0.05), but there was no difference between tillage regimes in cumulative xylose mineralized (Fig. S2, Table S4). These results indicate that tillage history impacted mineralization dynamics of C in the early period of xylose mineralization and throughout the incubation period for cellulose.

### Tillage alters bacterial community response to carbon inputs

**Fig 4:**
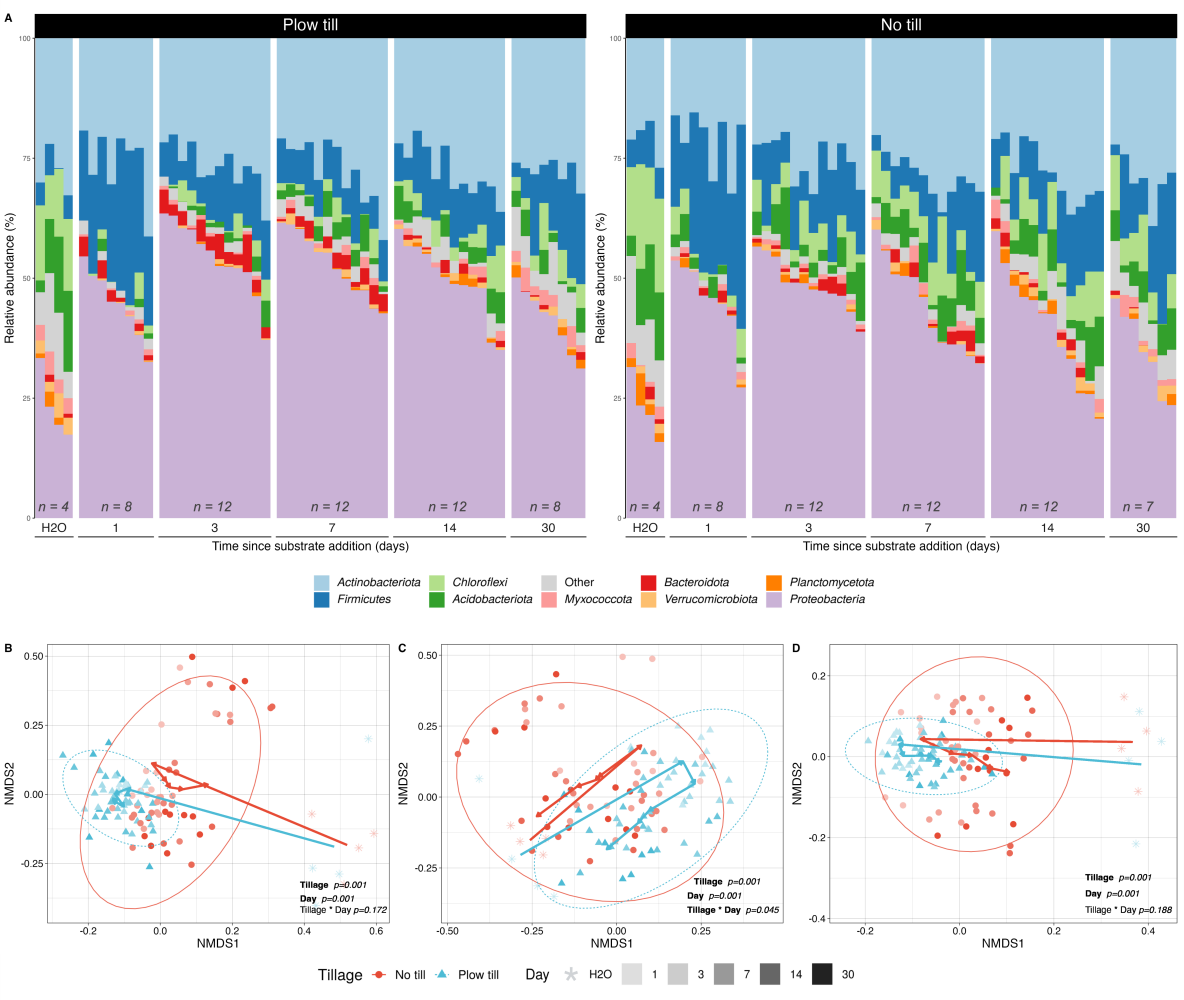
Bacterial communities differ in their response to carbon (^12^C + ^13^C) across tillage history and time. Relative abundance of bacterial phyla in microcosm bulk soil over time (**A**) indicates a proliferation of *Proteobacteria* relative to other taxa following C addition in both tillage regimes. Microbial community structure, including Bray-Curtis (**B**), unweighted UniFrac (**C**), and weighted UniFac (**D**) distances, varied according to days since C addition and tillage history. Vectors indicate the directional shift of community composition over time following C addition. The contribution of tillage and days since C addition to community structure was determined via PERMANOVA analysis in vegan (Table S6). Sample sizes of destructively harvested microcosms (^12^C + ^13^C) at each timepoint are indicated in (**A**).

We assessed temporal variation in bacterial community composition following carbon input to soil microcosms. At the start of the incubation, bacterial community membership in the microcosms was dominated by ASVs assigned to *Proteobacteria* and *Actinobacteria*. Both *Proteobacteria* and *Firmicutes* increased substantially within 1 day after carbon input (Fig. 4A). NTH soils exhibited greater evenness than PTH soils (mixed effects ANOVA, *P* < 0.01), but no differences in richness, Shannon diversity, or Inverse Simpson diversity were observed (Table S5). Using Maaslin2, we identified a total of 83 ASVs that were differentially abundant with respect to tillage history after accounting for sampling day. Of these differentially abundant taxa, 56 ASVs were enriched in PTH microcosms and largely belonged to the phylum *Proteobacteria* (Fig. S3). Twenty seven ASVs had differentially higher abundance in NTH microcosms after accounting for sampling day, and these were derived from the phyla *Proteobacteria*, *Actinobacteria*, and *Firmicutes*.

Microcosm community structure exhibited significant changes in beta diversity in response to C addition that varied according to tillage regime and time (Table S6). Communities that received C inputs differed significantly from water-only controls throughout the 30-day incubation, suggesting that time since C addition was stronger than tillage legacy in driving community composition (Fig. S4A and B). PERMANOVA analyses likewise indicated that time since C addition had roughly two- and four times greater explanatory power than tillage legacy for Bray-Curtis and weighted UniFrac beta diversity, respectively (Fig. 4B and D; Table S6). Differences in community structure between treatment and control were smaller, though still significant, when beta diversity was assessed using unweighted UniFrac distance (Fig. 4C; Fig. S4C). We also observed more dispersion in NTH communities than PTH communities throughout the incubation (Fig. 4B through D; PERMDISP *P* < 0.001 for weighted Unifrac and Bray-Curtis), suggesting that C addition had a stronger impact on community structure over time in NTH than in PTH.

### Bacterial carbon assimilation differs by tillage regime

**Fig 5:**
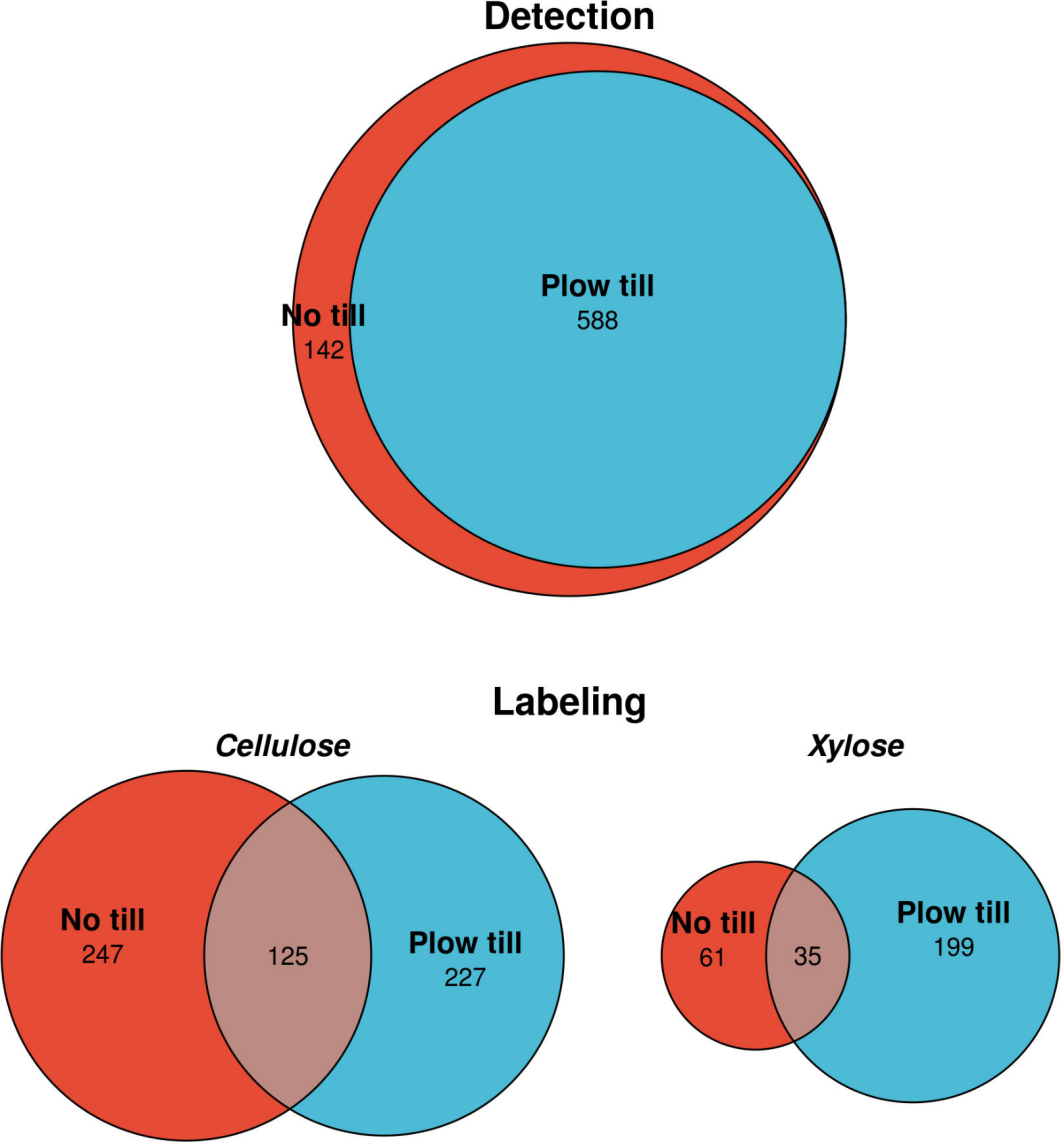
Long-term tillage regimes result in differential C assimilation by bacterial taxa. More xylose assimilating ASVs were detected in plow-till soils, while a similar number of cellulose-assimilating ASVs were detected in both tillage regimes. While the majority of ^13^C-labeled taxa were present in both land uses (detection), tillage determined patterns of ^13^C labeling, indicating active incorporation of labeled substrates. The figure includes incorporators identified across all DNA-SIP experiments.

To probe differences in bacterial C assimilation across tillage regimes, we employed multiple-window high-resolution DNA-SIP (MW-HR-SIP) (49). ASVs that incorporated ^13^C into their DNA from either ^13^C-xylose or ^13^C-cellulose were identified based on significant shifts in buoyant density relative to microcosms receiving ^12^C carbon. We identified 730 unique ASVs that assimilated ^13^C into their genomes. Of these, 468 ASVs were labeled in NTH microcosms, while 586 were labeled in PTH microcosms (Fig. 5). We found a similar number of ^13^C-cellulose incorporators in both soils (372 in NTH, 352 in PTH), but approximately 2.4 times more unique taxa incorporated ^13^C-xylose in PTH soils relative to NTH soils (234 in PTH and 96 in NTH). Strikingly, PTH soil contained 102 dual-labeled taxa compared to six incorporators in NTH that assimilated both ^13^C-cellulose and ^13^C-xylose. Furthermore, only 337 of the 730 incorporator ASVs were detected in unfractionated DNA from soil microcosms. DNA-SIP can detect rare taxa that often go undetected in standard DNA sequencing approaches; hence, many taxa (53.8%) participating in C cycling were present below the detection threshold in unfractionated microcosm communities.

**Fig 6:**
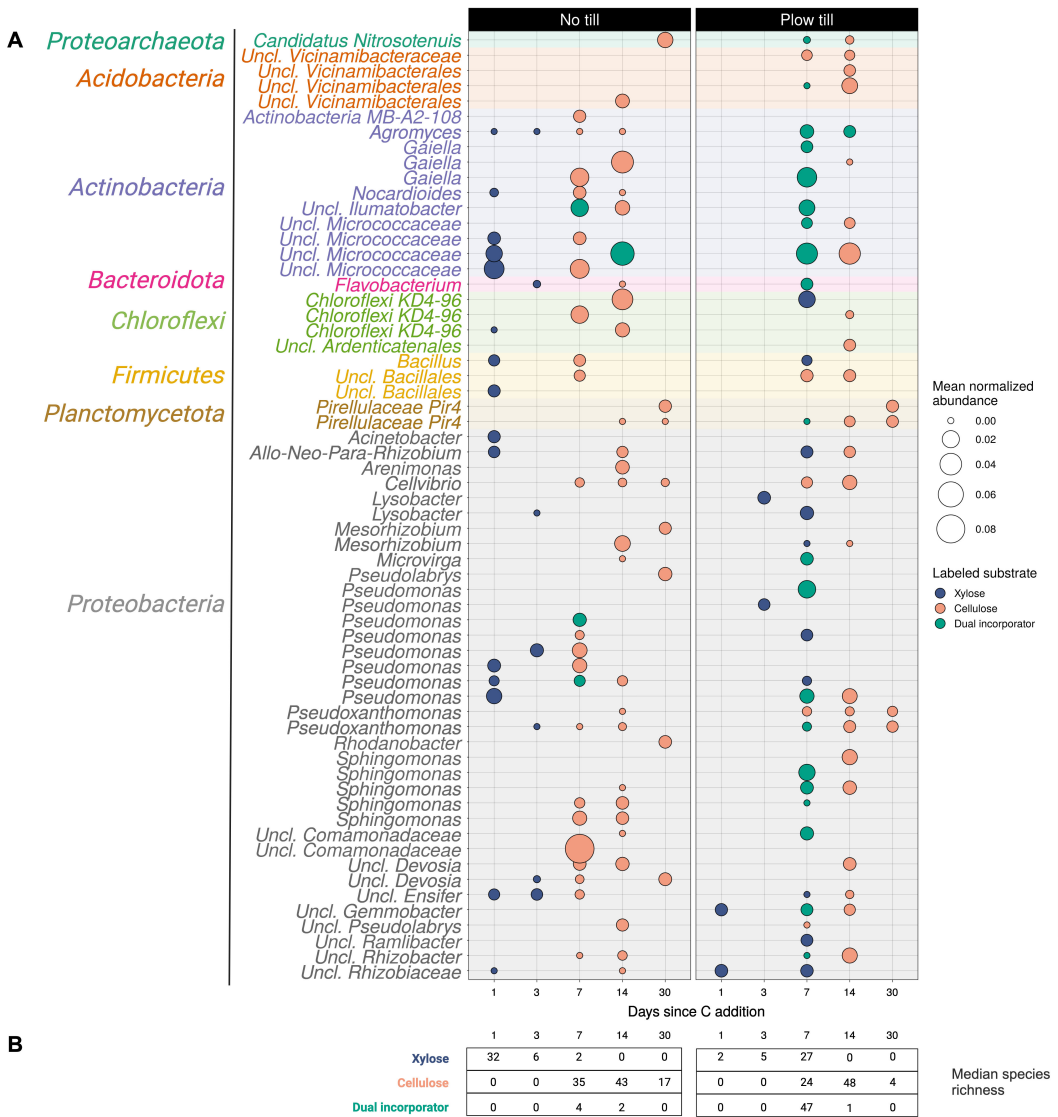
Growth and diversity of incorporator taxa differ by tillage regime. The top 35 most abundant incorporator ASVs from each tillage regime (**A**), grouped by phylogenetic relatedness and color coded by phylum, demonstrated earlier growth responses to xylose in no till relative to plow till. Point size represents the mean normalized abundance of incorporator ASVs across all microcosms that were identified via isopycnic centrifugation. Peak cellulose assimilation occurred between 7 and 14 days after C addition across both tillage histories. The median observed species richness of incorporator ASVs (**B**) differed with respect to tillage history, time, and substrate (Table S7). Plow till C assimilation was largely characterized by dual labeling of ASVs by both ^13^C-xylose and ^13^C-cellulose.

*Proteobacteria* such as *Pseudomonas* were particularly prevalent C incorporators, accounting for 48.7% of all labeled taxa detected in unfractionated microcosm communities (Fig. 6A). The most abundant incorporators in both tillage regimes derived from the phylum *Actinobacteria*, with unclassified *Micrococcaceae* assimilating ^13^C-cellulose in NTH soil on days 7 and 14, and a similar group assimilating both ^13^C-xylose and ^13^C-cellulose in PTH soil on day 7. Unclassified *Comamonadaceae* assimilated ^13^C-cellulose and were highly abundant in NTH soil but not in PTH soil (Fig. 6A). Incorporator diversity (Faith’s phylogenetic diversity and species richness) varied with respect to tillage, days since C addition, and their interaction (Table S7). Early NTH xylose incorporators demonstrated higher diversity immediately after substrate addition on day 1 (Fig. S5). PTH xylose incorporators were most diverse by day 7, coinciding with higher abundance and dual labeling with cellulose (Fig. 6A and B). Late cellulose incorporators were more diverse in NTH than PTH microcosms by day 30 (Fig. S5). Temporal patterns in incorporator diversity largely mirrored detection of labeling and normalized abundances of incorporators in microcosm communities.

### Incorporator growth dynamics differ by tillage and carbon source

**Fig 7:**
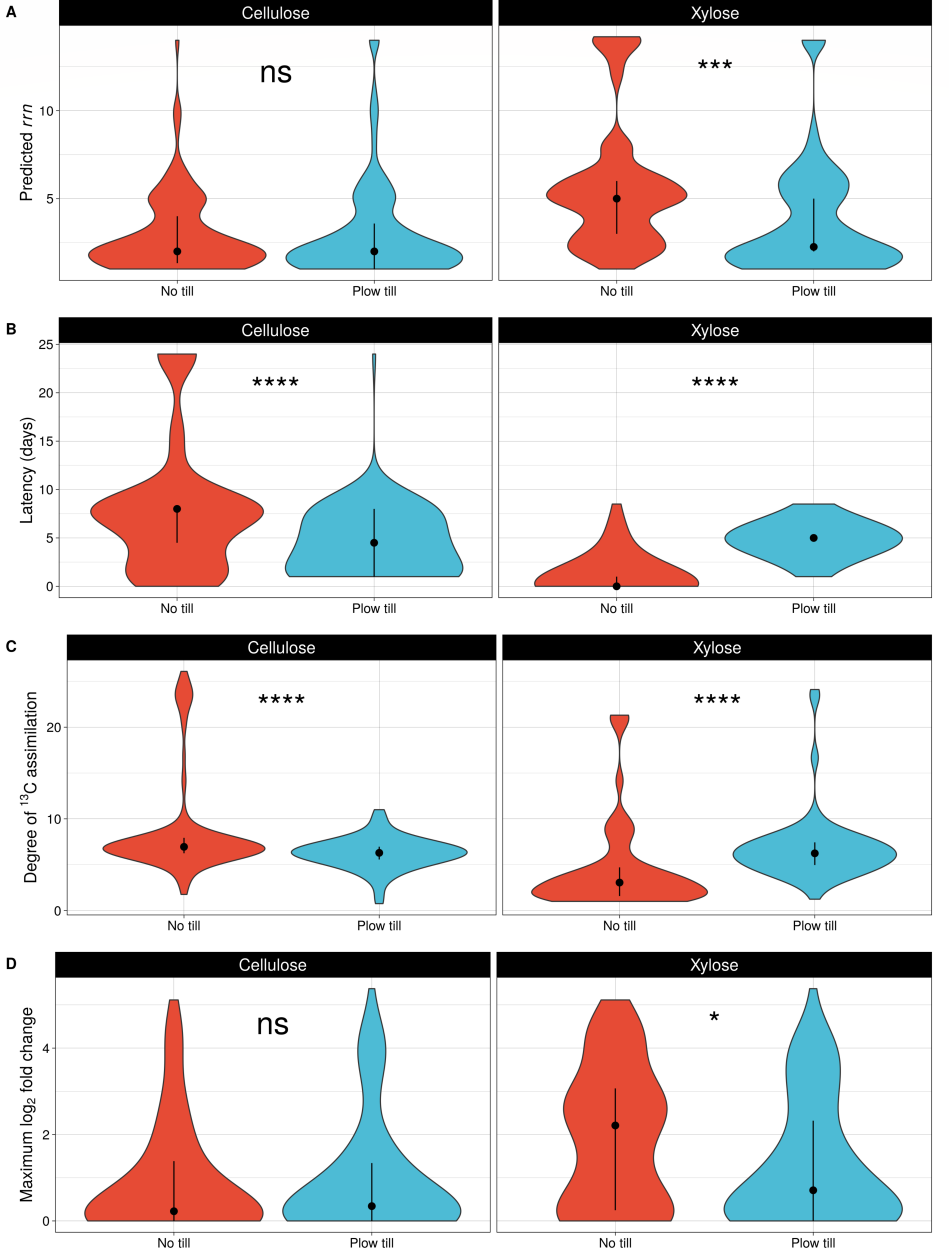
Predicted 16S rRNA copy number (**A**), latency (**B**), degree of labeling (**C**), and maximum log_2_-fold change (**D**) of incorporator ASVs differ with respect to tillage history and carbon substrate. The plot represents trait data for dereplicated incorporator ASVs that were present in bulk microcosm communities (NTH cellulose: 121 ASVs; NTH xylose: 51 ASVs; PTH cellulose: 124 ASVs; PTH xylose: 102 ASVs). Points indicate median values, and error bars denote the interquartile range. Significant differences (*P*-value < 0.05) between incorporator ASVs from different tillage histories were determined by Wilcoxon rank-sum tests.

We hypothesized that 42 years of tillage would alter C assimilation and growth dynamics due to selective pressures associated with disturbance (31). For each incorporator ASV present in rarefied microcosm communities, we calculated the predicted ribosomal RNA copy number (*rrn*) of each incorporator ASV due to its value as an indicator of life history, predictive of lag time (60) and translational power (23). Xylose incorporators that were present in rarefied microcosm communities had a median *rrn* of 5, while cellulose incorporators had a median *rrn* of 3 (Wilcoxon rank-sum test, *P* < 0.001; Fig. S6). Cellulose incorporator *rrn* did not differ by tillage regime although xylose incorporator *rrn* was higher in NTH (median *rrn* of 5) than in PTH (median *rrn* of 2.6; Wilcoxon rank-sum test, *P* < 0.001; Fig. 7A).

C assimilation and growth dynamics differed among incorporators according to tillage regime, predicted *rrn*, and substrate. Incorporator latency, the difference between detection and peak mineralization activity, differed by C substrate and tillage (Wilcoxon rank sum test, *P* < 0.001; Fig. 7B). If an organism is labeled after peak mineralization activity has occurred, it is more likely to have acquired ^13^C from prior microbial processing rather than directly from the labeled substrate (24). Xylose incorporators that were present in rarefied microcosm communities exhibited an earlier growth response with low latency (0 days) in NTH while exhibiting a lagged growth response with high latency (5 days) in PTH. Xylose labeling in NTH soils occurred mostly (69.6%) on day 1 and was dominated by high *rrn* ASVs (Fig. 6A and B; Fig. S7). In PTH soils, 90.2% of xylose labeling occurred 6 days later, on day 7, outside of peak mineralization activity, and these taxa were dominated by a diverse mixture of high and low *rrn* ASVs (Fig. S7). In PTH soils, many of these late day 7 xylose-C incorporators were also able to assimilate cellulose-C (Fig. 6A and B). Low latency of cellulose assimilation in PTH (4 days) contrasted with high latency of cellulose assimilation in NTH (8 days). Long-term tillage, therefore, resulted in a lagged growth response of xylose incorporators and dual labeling of taxa that had a mix of low and high *rrn* copy number. In contrast, NTH soils exhibited early assimilation of xylose-C by high *rrn* copy number bacteria and delayed assimilation of cellulose-C by taxa unable to access xylose.

Next, we examined the degree of ^13^C assimilation among incorporators across the two tillage regimes and C substrates (see Materials and Methods). While not equivalent to atom % enrichment, the degree of ^13^C assimilation explains substantial variation in ^13^C enrichment and depends both on abundance in “heavy” fractions in addition to genomic GC content (59). NTH cellulose incorporators exhibited, on average, 40.5% higher labeling than PTH cellulose incorporators (Wilcoxon rank-sum test, *P* < 0.001). Conversely, PTH xylose incorporators exhibited an average of 47.4% higher labeling than NTH xylose incorporators (Wilcoxon rank-sum test, *P* < 0.001; Fig. 7C). Differences in the degree of ^13^C assimilation, consistent with differences in latency, indicate that tillage history altered the metabolic path of C through the soil community.

We also examined maximum log_2_-fold change (max l2fc), which is an indicator of dynamic growth potential associated with ruderal life history strategies (30). Cellulose incorporators did not differ by max l2fc with respect to tillage regime. In contrast, the max l2fc of xylose incorporators was threefold higher in NTH than in PTH (Wilcoxon rank-sum test, *P* = 0.03; Fig. 7D). Our results demonstrate a higher potential for dynamic growth among xylose incorporators in NTH relative to PTH, suggesting that ruderals dominated xylose metabolism in NTH to a greater extent than in PTH.

Finally, we examined whether growth responses to C (latency, max l2fc, degree of ^13^C assimilation) were phylogenetically conserved with respect to tillage. C cycling responses exhibited little phylogenetic conservation beyond the genus level regardless of tillage regime (Fig. S8). Growth responses to C among incorporators were more strongly related to phylogenetic distance in NTH soils (Spearman’s ρ = 0.17, *P* < 0.001) than in PTH soils (Spearman’s ρ = −0.07, *P* = 0.001).

## DISCUSSION

### Tillage altered carbon mineralization rates and total carbon mineralization

We found that a 42-year history of moldboard plowing significantly altered the mineralization dynamics of xylose and cellulose, two C substrates that are major components of plant cell walls and which differ in bioavailability. We show that no-till soils had higher mineralization rates than tilled soils for both xylose and cellulose (Fig. S1), with differences for xylose mineralization maximal within 5 days of substrate addition, and differences for cellulose mineralization persisting through all 30 days of the experiment (Fig. S2). These differences in mineralization rates correspond to wholesale differences in bacterial ^13^C-assimilation in tilled and no-till soils (Fig. 5 and 6). Our findings suggest that differences in bacterial C metabolism between tilled and no-tilled soils caused faster C mineralization of both xylose and cellulose in no-till soils relative to tilled soils.

Our results highlight the important role of historical disturbance in contributing to divergent C dynamics in managed ecosystems. Both soils were managed identically for 42 years, with tillage as the sole variable, resulting in significantly higher organic matter in NTH soils relative to PTH soils (32). NTH soils had higher total C than PTH soils (Table S1), likely because decomposition of intact maize roots was altered due to tillage. No-till legacies have been associated with higher rates of C stabilization due to the preservation of macro-aggregates which protect C from microbial activity (61) and reduced mechanical breakdown of plant residues. While tillage legacies altered SOC accumulation in the plots we used for soil incubations (32), our experimental design controlled for differences in initial labile C by imposing a 14-day pre-incubation in which CO_2_ production was monitored. Substrates were added only after respiration stabilized, and we did not find significant differences in C mineralization between tillage regimes in water-only controls, suggesting similar amounts of native bioavailable soil C during the experiment (Fig. S1). Therefore, we conclude that differential mineralization of xylose and cellulose is related to microbial growth dynamics arising from tillage legacy.

### Tillage-structured bacterial community responses to carbon

We show that tillage legacy altered bacterial community structure and response to carbon. Beta-diversity (Bray-Curtis and weighted/unweighted Unifrac) varied by tillage history and time since C addition. Differences in evenness between tillage regimes were explained by the differential abundance of specific taxa rather than differences in species composition of communities. Carbon input to tilled soil was accompanied by dynamic increases in the relative abundance of *Proteobacteria, Firmicutes,* and, to a lesser extent, *Actinobacteria* (Fig. 4A). These phyla all have been previously linked to legacies of disturbance (62, 63) or artificially imposed disturbance (64). Thus, tillage resulted in divergent responses to C within bacterial communities, likely related to the proliferation of disturbance-adapted taxa in tilled soils.

Plow-till microcosm communities were also more homogeneous (i.e*.,* less dispersed in community structure) than no-till communities. This observation concurs with prior literature that documents the homogenizing influence of disturbance on microbial communities (64). By inverting topsoil and disrupting soil physical structure, tillage may structure bacterial communities through homogenizing dispersal (11) and reducing microsite variation in community diversity associated with niche habitats in undisturbed soil aggregates (65–67). Given prior evidence, we expect that stochastic processes dominate bacterial community assembly in tilled soil (11, 68, 69). The impact of community assembly processes on carbon cycling has not previously been addressed, and it is unclear how stochasticity alters the C cycling function of bacterial communities. However, it is evident that the legacy impacts of tillage structured community-wide responses to C addition that were not explained by overall differences in diversity or biomass (assessed by DNA yield). If microbial biomass and diversity are lower in arable soils relative to other land uses (70–72), community assembly processes may have an outsized influence on carbon assimilation dynamics by impacting the successional state of the community.

### Tillage legacy resulted in streamlined processing of carbon

Our results indicate that the historical effect of tillage caused fundamental differences in bacterial growth dynamics and carbon mineralization relative to no-till soils. We hypothesized that a legacy of disturbance due to tillage would favor pulse-adapted taxa that preferentially consume high bioavailability substrates such as xylose. Although we identified a higher richness of xylose incorporators in PTH soils (Fig. S5), 90.2% of xylose-incorporating bacterial ASVs in PTH microcosms were not labeled until day 7, while peak xylose mineralization occurred on day 2. In addition, many taxa that assimilated C from xylose in PTH could also assimilate C from cellulose. In contrast, xylose C was assimilated rapidly in NTH soils (Fig. 6A), and few taxa in NTH were able to assimilate C from both xylose and cellulose. This implies that xylose metabolism in NTH microcosms was dominated by primary assimilation driven by high *rrn* growth adapted specialists, while tillage caused a lagged growth response characterized by downstream processing driven by a broader diversity of taxa which assimilated C from both xylose and cellulose. We expect that these differences in community growth dynamics are directly linked to the greater mineralization rates observed in no-till soils.

The results show that bacterial metabolism of xylose and cellulose proceeds through different channels in the no-till soils, and this specialization supports higher mineralization rates in NTH relative to PTH. The difference in xylose assimilation dynamics between PTH and NTH could be driven by differences in SOC stocks with respect to tillage. NTH soils have higher SOC than PTH, and this could support greater microbial competition and specialization, which might explain why xylose and cellulose incorporators exhibited vastly different assimilation dynamics and diversity.

In contrast, tillage resulted in the dual assimilation of both xylose and cellulose coupled with lower mineralization activity. ^13^C-cellulose labeling in plow-till soil co-occurred with lagged ^13^C-xylose labeling on day 7, suggesting a single channel for carbon metabolism. In addition to higher functional redundancy with fewer taxa involved in C cycling, the dual-labeling we observed in PTH could indicate metabolic dependency among labeled microorganisms. Dual labeling in PTH was highest among members of *Actinobacteria* and *Proteobacteria*, which include potentially non-celluloytic taxa that assimilated byproducts of cellulose metabolism or molecules released from the biomass of primary incorporators (73, 74).

We did not find evidence to support a predictive role for incorporator phylogeny in explaining differences in C cycling across the two tillage regimes. Previous DNA-SIP experiments found that bacterial incorporation of bioavailable C is phylogenetically constrained, with *Actinobacteria* demonstrating consistent growth in response to glucose across multiple ecosystems (74–76). We found that incorporator phylogeny was a relatively poor predictor of C incorporation traits (24), and the relationship between phylogenetic distance and functional distance was especially marginal in plow-till soil (Fig. S7).

### Growth responses inclusive of life history drive bacterial carbon metabolism

We predicted that a 42-year history of tillage would favor the proliferation of growth-adapted, ruderal, taxa owing to a high disturbance environment (31). In agreement with previous reports (24), we found that predicted *rrn* of labeled ASVs was a reliable indicator of carbon use preference with respect to substrate bioavailability. Xylose, which is highly soluble and readily available for diffusive transport, was preferentially assimilated by high *rrn* organisms (25). Cellulose, which is insoluble, was assimilated by low *rrn* organisms in both tillage regimes (Fig. S6 and S7). We interpret the lower median *rrn* of cellulose incorporators relative to xylose incorporators as evidence that cellulose metabolism was predominantly conducted by taxa adapted for resource acquisition in both tillage regimes (73).

**Fig 8:**
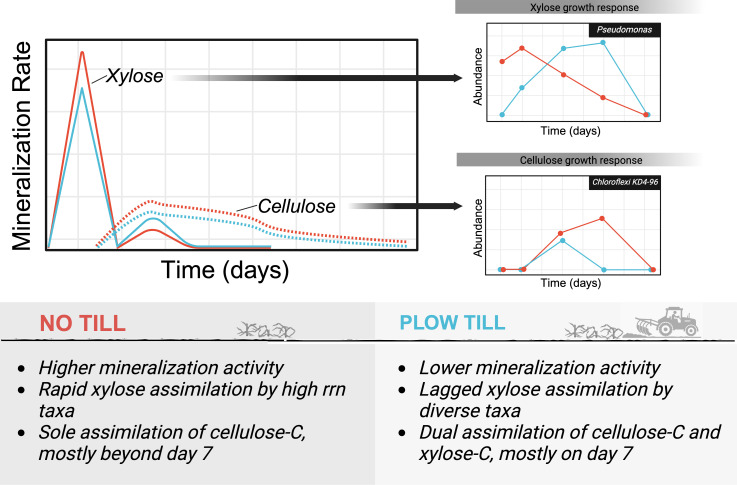
Growth responses of incorporator taxa contribute to differences in mineralization activity observed for each carbon substrate by tillage regime, indicated by color. Plow-till soils were associated with the co-metabolism of cellulose and xylose through a single carbon channel that was growth-limited, resulting in lower mineralization rates for both substrates. Ruderal xylose incorporators demonstrated a delayed growth response in plow-till soils, with maximum abundance and labeling occurring well after peak xylose mineralization. Many cellulose incorporators in plow-till soils were dual labeled with xylose. In contrast, no-till cellulose incorporators had high latency and generally did not assimilate xylose, suggesting that substrates of differing bioavailability were processed through distinct channels that were not growth-limited. Average growth curves of incorporators belonging to the *Pseudomonas* and *Chloroflexi KD4-96* genera are representative of this general trend. We hypothesize that tillage altered the successional state of bacterial communities, differentially impacting the growth responses of ruderal C-cycling microorganisms.

Concurrent with higher *rrn,* NTH xylose incorporators demonstrated significantly lower latency and higher max l2fc (Fig. 7) than PTH xylose incorporators. These results lend support for a distinct, growth-adapted response to xylose incorporators in no-till that was largely absent in plow-till (Fig. 8). Tillage did not alter C mineralization dynamics solely by favoring the proliferation of ruderal organisms over competitor taxa. Rather, the observed differences in xylose and cellulose mineralization were likely driven by lagged growth of high *rrn* ruderal incorporators in PTH soil. We hypothesize that disturbance altered the successional state of microbial communities in tilled soil, contributing to lower initial abundance of active C-cycling bacteria and lagged ruderal growth. This resulted in separate channels for xylose and cellulose processing in no-till, but a single growth-limited channel in plow-till.

We expect that, as with bacteria, disturbance regimes will differentially favor fungi with ruderal life history strategies, resulting in cross-kingdom interactions that have consequences for carbon cycling. Indeed, analysis of fungal ITS sequences reveals that tillage shifts the structure of fungal communities at this site, and *Ascomycota* in PTH relative to NTH (Fig. S9 and S10). Hence, one explanation for the high latency we observed in ^13^C xylose assimilation in PTH is that bacterial assimilation of xylose in PTH could have been secondary to fungal processing. If true, this hypothesis would provide an explanation for the frequency of dual incorporators in PTH, suggesting that most bacterial C assimilation was secondary to fungal processing in the tilled soils, but not in NTH. Fungal-bacterial interactions are likely to play an important role in determining flows of C in soil, and it is possible that many of the differences we observed in bacterial assimilation dynamics were driven by changes in fungal-bacterial interactions caused by tillage.

In summary, C metabolism in plow-till soils was characterized by diverse taxa that incorporated C from both cellulose and xylose but only after a substantial growth lag. Meanwhile, C metabolism in no-till soils was characterized by separate channels for the assimilation of C from xylose and cellulose (Fig. 8).

### Conclusion

We found that long-term tillage alters bacterial growth responses with functional implications for C cycling. Tilled soil exhibited lagged assimilation of ^13^C-xylose into bacterial DNA, which corresponded to significantly lower rates of xylose mineralization relative to no-till soil. In addition, tilled soils exhibited many taxa that assimilated both ^13^C-cellulose and ^13^C-xylose while no-till soils had few such dual incorporators. This pattern of dual incorporation corresponded with lower rates of cellulose mineralization in tilled relative to no-tilled soil. While there are several potential mechanisms to explain these results, our findings show that tillage fundamentally alters bacterial C cycling in soils by altering both mineralization rates and the dynamics of in-vivo processing. Understanding the impacts of microbial growth dynamics on the fate of carbon will improve future efforts to predict changes in soil organic carbon stocks resulting from management.

## Data Availability

Processed sequences from fractionated and unfractionated samples were deposited in the NCBI Short Read Archives (BioProject PRJNA1170979). All scripts used in processing and analyzing the sequence data can be accessed at https://github.com/schaedem/chazy_sip.

## SUPPLEMENTAL MATERIAL

Supplemental material aem.00933-25-s0001.docx Supplemental methods, Tables S1 to S7, and Fig. S1 to S10.

ASM does not own the copyrights to Supplemental Material that may be linked to, or accessed through, an article. The authors have granted ASM a non-exclusive, world-wide license to publish the Supplemental Material files. Please contact the corresponding author directly for reuse.
