# Supplementary material for: Long term tillage regime alters bacterial assimilation of xylose and cellulose
Source: Appl Environ Microbiol. 2025 Aug 6;91(9):e00933-25. doi: 10.1128/aem.00933-25 (PMC12442353; doi:10.1128/aem.00933-25)
Supplement: Supplemental material — Supplemental methods, Tables S1 to S7, and Fig. S1 to S10. [file aem.00933-25-s0001.docx]

**Supplemental Methods**

**Microcosm Set-Up**

Microcosms were set up in 250 mL Erlenmeyer flasks. Ten grams dry weight soil were placed in the flasks and sealed with butyl rubber stoppers to prevent moisture loss. Dry weight was determined by gravimetric soil moisture measurements for three technical replicates within each tillage treatment and biological replicate (1). Microcosms were pre-incubated for 2 weeks until the production of CO_2_ stabilized following disruption due to sieving, as assessed by GC-MS (Shimadzu QP2010S GC-MS plumbed with Carboxen-1010 PLOT column, St. Louis, MO) analysis.

Carbon substrates for microcosm enrichment were chosen based on the composition of corn stover (2) due to the long-term cropping history of corn in the field site where soils were collected. The substrate solution contained cellulose (0.889 mg C g^-1^ soil), xylose (0.451 mg C g^-1^ soil), arabinose (0.062 mg C g^-1^ soil), mannose (0.034 mg C g^-1^ soil), galactose (0.042 mg C g^-1^ soil), and lignin (0.591 mg C g^-1^ soil). The remaining 18% mass was composed of amino acids (Teknova, #C0705) and Murashige and Skoog basal salt mixture (Millipore Sigma, #M5524). The substrate solution had a C:N ratio of 10, which was comparable to the C:N ratio of the soils used in the experiment. Matching C:N ratios between the substrate solution and soil allowed us to observe mineralization activity of added substrates while limiting the confounding effects of C immobilization. All components of the solution, except for the insoluble C substrates cellulose and lignin, were added in dissolved form at 50% of the water holding capacity of the soil. The two ^13^C treatments substituted 99% ^13^C enriched cellulose or xylose. Both ^13^C and ^12^C cellulose used in the stable isotope probing (SIP) microcosms were prepared using *Gluconoacetobacter xylinus* as described previously (3). The ^12^C control treatments contained carbon with a natural abundance of ^13^C. Water-only control microcosms were treated with an equivalent amount of water and basal salts as other treatments to serve as a control for moisture effects.

Microcosms (n = 112) were prepared using soil derived from field replicates (n = 4). Unlabeled control microcosms and water only controls were destructively sampled five times at 1, 3-, 7-, 14-, and 30-days following substrate addition, resulting in 20 microcosms each for till and no-till soil (4 replicates, 5 timepoints). Microcosms receiving ^13^C cellulose were sampled on days 3, 7, 14, and 30, while ^13^C xylose microcosms were sampled on days 1, 3, 7, and 14 (Figure 1). This resulted in a total of 16 microcosms for each tillage x substrate treatment. We limited our destructive sampling times to expected windows of substrate mineralization activity for xylose and cellulose. These sampling timepoints were selected because results from this and previous studies (3–6) indicate that xylose is completely mineralized by day 14 while appreciable cellulose mineralization occurs only after 3 days. A total of 8 water-only microcosms were prepared as controls with 4 replicates from each tillage regime. Water-only controls were used to monitor baseline mineralization in the absence of carbon and were destructively sampled at the end of the experiment on day 30. Soil from harvested microcosms was stored at –80°C until DNA extraction was performed. Day 30 soils were sub-sampled for isotopic analysis (UC Davis Stable Isotope Facility) and determination of pH, total C, and total nitrogen (N). Soil pH was determined using a 1:1 soil-water slurry method. Total C and N were measured using oven dried, ground samples via a LECO Treu Mac CN-2000 elemental analyzer (LECO Instruments, Lansing, MI) as previously described (1).

**DNA Extraction**

We extracted DNA from bulk (unfractionated) microcosms using 2 x 0.25 g of soil from all replicates of each isotope x day x soil combinations (112 samples). We used a modified Griffiths phenol-chloroform extraction procedure (7), in which cells were first lysed by 1 minute of bead-beating at 5.5 m s^-1^ in 2 mL lysis tubes filled with 0.5 g of 0.1 mm silica/zirconia beads, 0.5 mL extraction buffer (240 mM phosphate buffer with 0.5% N-lauryl sarcosyl), and 0.5 mL of phenol-chloroform-isoamyl alcohol (25:24:1). After lysis, 85 µL of NaCl (5 M) and 60 µL of a mixture including hexadecyltriammonium bromide (CTAB, 10%) and 0.7 M NaCL were added to the tube. The tube was then vortexed, chilled on ice for 1 minute, and centrifuged at 16,000 x g for 5 minutes at 4°C. The top aqueous layer was transferred to a new tube and placed on ice. The pellet was re-extracted following a similar procedure, with the aqueous layer again removed and combined with the first aqueous layer. These combined layers were washed with 1 mL chloroform : isoamyl alcohol (24:1) and DNA was precipitated with 2 volumes of polyethylene glycol solution (30% PEG 8000, 1.6 M NaCl) at 4°C. Precipitate was collected by centrifugation at 16,000 x g for 30 minutes at 4°C, the supernatant was removed, and the pellets washed with 1 mL of 70% EtOH. Finally, dried pellets were resuspended in 50 µL TE and stored at –20°C. Following extraction, DNA extracts from unfractionated samples were cleaned using illustra™MicroSpin™ G-50 columns (GE Healthcare; Buckinghamshire, UK; 27-5330-02) and magnetic bead purification (Agencourt AMPure XP purification; Beckman Coulter; Brea, CA; A63880), according to manufacturer protocols.

DNA for isopycnic centrifugation was extracted from 4 technical replicates of 0.25 g of soil, following the phenol-chloroform procedure outlined above. A subset of microcosm biological replicates was selected for isopycnic centrifugation: replicate 4 across all destructive sampling timepoints for ^13^C cellulose/^13^C xylose/^12^C controls, replicates 2 and 3 for ^13^C/^12^C xylose on day 3, and replicates 2 and 3 for ^13^C/^12^C cellulose on day 30. Technical replicates of DNA extractions were pooled and selected for a size of 4 – 14 kb with a Blue Pippen Prep machine (Sage Science, Beverly, MA) according to the manufacturer’s protocol.

**Stable isotope probing and isopycnic centrifugation**

Isopycnic centrifugation was performed for a total of 42 samples: all isotope treatment x day x tillage samples from replicate 4 (n = 26), day 3 cellulose samples of both isotope and tillage treatments from replicates 2 and 3 (n=8), and day 30 cellulose treatments from replicates 2 and 3 (n=8). We prepared isopycnic gradients as described previously (4, 5, 8). Briefly, 6 µg of size-selected DNA from each set of pooled samples was added to the density gradient solution (1.69 g ml^-1^) in a 4.7 mL polypropylene tube (Beckman Coulter, Brea, CA). The gradient solution was made from a concentrated stock of CsCl (1.9 g mL^-1^) and diluted in a buffer solution containing 15mM Tris-HCl, 15 Mm EDTA, and 15 mM KCl to reach the target density of 1.69 g mL^-1^. Sample tubes were centrifuged at 55,000 rpm for >66 hours at 20 °C on an Optima MAX-E ultracentrifuge (Beckman Coulter; Brea, CA) with a TLA-1 10 fixed-angle rotor.

Following centrifugation, 100 µl density DNA fractions were collected from the bottoms of the polypropylene tubes using syringe pump-mediated water displacement at a rate of 15 µl s^-1^ (9). Fractions were collected in a deep-well 96-well plate (Corning, Tewksbury, MA). Immediately after each fraction was collected, we measured its refractive index (R_i_) using a Reichart AR200 refractometer . The R_i_ of each fraction was corrected by subtracting the R_i_ of the gradient buffer and water (R_i corrected_ = R_i observed –_ R_i buffer –_ R_i water_) and used to calculate the buoyant density of the DNA gradient:

Density (g mL^-1^) = a R_i corrected_ - b

In the above equation, a and b are coefficient values of 10.9276 and 13.593, respectively, for CsCl at 20°C (12). Fractions in the range of 1.673 -1.774 g mL^-1^ were chosen for sequencing. This density range represents DNA segments that range in GC content from 13.5 - 80% plus an additional 0.036 g mL^-1^ for ^13^C labeling. An average number of 23 fractions per gradient tube were used, resulting the preparation of 979 fractions for sequencing.

Collected fractions intended for sequencing were desalted using the Agencourt AMPure XP purification kit according to the manufacturer’s protocol (Beckman Counter, Brea, CA). Finally, we quantified purified DNA using the Quant-IT PicoGreen dsDNA assay (Life Technologies, Grand Island, NY). Fluorescence was measured using a FilterMax F5 plate reader (Molecular Devices, Sunnyvale, CA).

**16S rRNA library preparation and sequencing**

We performed amplicon sequencing of the v4 region of 16S rRNA gene across all unfractionated (n=112) and fractionated (n=979) samples. After fractionation and desalting, DNA SIP fractions within the density range of 1.673 - 1.774 g/mL were sequenced. This density range represents DNA segments that range in GC content from 13.5-80% plus an additional 0.036 g/mL for ^13^C labelling. An average number of 23 fractions per gradient column were used, resulting in the preparation of 979 fractions for sequencing.

The V4 hypervariable region of the 16S rRNA gene was targeted with the 515f / 806r primer set (13). Identical protocols were followed for 16S rRNA library preparation for fractionated and unfractionated samples, except for the addition of 1.25 ul Bovine Serum albumin (BNSA, New England Biolabs) to the PCR reactions of unfractionated samples. The volume of PCR reactions was 25 ul, consisting of 12.5 ul Q5 High Fidelity Hot Start PCR Mastermix (New England Biolabs), 2.5 ul combined forward and reverse barded primer at 10 uM, 5 ng template DNA, and 0.625 ul Picogreen reagent (Life Technologies, Grand Island, NY). Picogreen was added to enable visualization of reaction process on a qPCR machine. Triplicate PCR samples were normalized using a SequalPrep Normalization kit (Invitrogen), pooled, and concentrated to 5 ng/ul. Amplicon libraries were size-selected at 400-600 bp via gel excision and extracted using Wizard SV Gel and PCR Clean-Up kit (Promega). Pooled amplicon libraries were submitted for sequencing at the Cornell Core Facility in Ithaca, NY. Samples were run on an Illumina MiSeq using V2 chemistry with 2 x 250 bp read length.

**Normalized abundance calculation for incorporator ASVs**

$$ASV relative abundance \times\frac{ASV rrn}{community rrn} \times DNA yield (ng {ul}^{-1})$$

**Supplemental Results**

**Supplemental Table 1. Soil characteristics of the long-term tillage experiment in Chazy, NY.** All measurements, except moisture, were taken in September 2014. Moisture was averaged over 11 sampling timepoints from July 2014 to November 2015.

| **Tillage** | **%C** | **%N** | **C:N** | **pH** | **% Moisture** | **DNA Yield (ng/ul)** |
| --- | --- | --- | --- | --- | --- | --- |
| **No-till** | 2.13 ± 0.39 | 0.18 ± 0.04 | 11.58 ± 0.32 | 6.89 ± 0.93 | 16.5 ± 2.47 | 56.0 ± 8.99 |
| **Till** | 1.50 ± 0.26 | 0.10 ± 0.02 | 14.36 ± 1.12 | 7.74 ± 0.05 | 16.5 ± 6.59 | 75.9 ± 26.9 |

**Supplemental Table 2. Linear mixed effects model results evaluating the contribution of tillage regime, sample day, and their interaction on mineralization rates (mg CO_2_ hr^-1^) of total carbon (^12^CO_2_ + ^13^CO_2_) and individual substrates (^13^C-xylose or ^13^C-cellulose).** Field replicate was included as a random effect in all models.

| **Substrate** | **Fixed Effect** | ***df*** | ***F*-value** | ***p*-value** |
| --- | --- | --- | --- | --- |
| **Total carbon** | Tillage | 1 | 21.1883 | <0.0001 |
|  | Day | 19 | 492.5228 | <0.0001 |
|  | Tillage:Day | 19 | 5.3643 | <0.0001 |
| **Xylose** | Tillage | 1 | 1.0044 | 0.319 |
|  | Day | 11 | 178.4375 | <0.0001 |
|  | Tillage:Day | 11 | 3.9743 | <0.001 |
| **Cellulose** | Tillage | 1 | 11.0489 | 0.001 |
|  | Day | 17 | 28.1886 | <0.0001 |
|  | Tillage:Day | 17 | 3.4327 | <0.001 |

**Supplemental Table 3. Linear mixed effects model results evaluating the contribution of tillage regime, sample day, and their interaction on daily cumulative mineralization (mg CO_2_ hr^-1^) of total carbon (^12^CO_2_ + ^13^CO_2_) and individual substrates (^13^C-xylose or ^13^C-cellulose).** Field replicate was included as a random effect in all models.

| **Substrate** | **Fixed Effect** | ***df*** | ***F*-value** | ***p*-value** |
| --- | --- | --- | --- | --- |
| **Total carbon** | Tillage | 1 | 28.706 | <0.0001 |
|  | Day | 19 | 66.763 | <0.0001 |
|  | Tillage:Day | 19 | 0.4238 | 0.99 |
| **Xylose** | Tillage | 1 | 23.5167 | <0.0001 |
|  | Day | 11 | 858.4579 | <0.0001 |
|  | Tillage:Day | 11 | 2.6409 | <0.01 |
| **Cellulose** | Tillage | 1 | 101.4207 | <0.0001 |
|  | Day | 17 | 264.4105 | <0.0001 |
|  | Tillage:Day | 17 | 2.3875 | <0.01 |

**Supplemental Table 4. Average cumulative carbon mineralized (mg CO_2_) from total carbon (^12^CO_2_ + ^13^CO_2_) and individual substrates (^13^C-xylose or ^13^C-cellulose) by experimental endpoints.** Microcosm headspace was analyzed for xylose mineralization up to day 14 and for cellulose up to day 30 Superscripts indicate significant differences (p<0.05) according to post-hoc means separation from linear mixed models comparing cumulative mineralization from microcosm replicates between tillage regimes (Supplemental Table 3).

| **Tillage** | **Substrate** | **Day** | **mg CO_2_** | **SD** |
| --- | --- | --- | --- | --- |
| No till | Total carbon | 14 | 9.03 | 1.90 |
|  |  | 30 | 11.60 | 2.02 |
|  | Xylose | 14 | 1.89 | 0.10 |
|  | Cellulose | 30 | 3.12^a^ | 0.28 |
| Plow till | Total carbon | 14 | 7.93 | 1.86 |
|  |  | 30 | 9.73 | 1.92 |
|  | Xylose | 14 | 1.87 | 0.06 |
|  | Cellulose | 30 | 2.81^b^ | 0.16 |

**Supplemental Table 5. Tillage regime and days since carbon addition drive differences in bacterial community evenness, but not other measures of alpha diversity.** Linear mixed models were used to determine the contribution of tillage and days since C addition to bulk microcosm alpha diversity, with replicate included as a random factor. Alpha diversity measures were normally distributed according to Shapiro-Wilkes tests and values from rarefied bulk microcosm soil (irrespective of isotopic label) were used as response variables for the models. The significance of fixed effects was determined by analysis of variance in base R.

|  | **Fixed Effect** | ***df*** | ***F*-value** | ***p*-value** |
| --- | --- | --- | --- | --- |
| **Shannon** | Tillage | 1 | 90.053 | 0.98 |
|  | Day | 4 | 90.045 | 0.24 |
|  | Tillage:Day | 4 | 90.045 | 0.44 |
| **Pielou’s evenness** | Tillage | 1 | 8.4212 | <0.01 |
|  | Day | 4 | 3.7490 | <0.01 |
|  | Tillage:Day | 4 | 0.8983 | 0.47 |
| **Inverse Simpson** | Tillage | 1 | 1.4762 | 0.23 |
|  | Day | 4 | 2.0927 | 0.09 |
|  | Tillage:Day | 4 | 1.1512 | 0.34 |
| **Richness** | Tillage | 1 | 0.6539 | 0.42 |
|  | Day | 4 | 1.6012 | 0.18 |
|  | Tillage:day | 4 | 0.8880 | 0.47 |

**Supplemental Table 6. Tillage regime and days since carbon addition contribute to variation in bacterial community structure.** PERMANOVA analyses were conducted using *adonis2* in vegan (14).

|  | **Fixed Effect** | ***df*** | ***R^2^*** | ***p*-value** |
| --- | --- | --- | --- | --- |
| **Bray-Curtis** | Tillage | 1 | 0.06 | <0.001 |
|  | Day | 5 | 0.14 | <0.001 |
|  | Substrate:Day | 5 | 0.04 | 0.17 |
| **Weighted UniFrac** | Tillage | 1 | 0.07 | <0.001 |
|  | Day | 5 | 0.27 | <0.001 |
|  | Substrate:Day | 5 | 0.04 | 0.02 |
| **Unweighted UniFrac** | Tillage | 1 | 0.04 | <0.001 |
|  | Day | 5 | 0.11 | <0.001 |
|  | Substrate:Day | 5 | 0.05 | 0.05 |

**Supplemental Table 7. Incorporator diversity varies with respect to tillage regime, days since C addition, and their interaction.** Incorporator species richness and phylogenetic diversity demonstrated non-normal distributions that were not corrected by log or square-root transformations. We therefore modeled the effects of tillage regime and days since C addition on incorporator diversity with generalized linear mixed models that included microcosm replicate as a random effect. Models were fit with *glmer* from the lme4 package (15). Models for species richness were fit with a Poisson distribution (family = poisson) while models for phylogenetic diversity were fit with a gamma distribution (family = gamma). The significance of fixed effects was determined with type III Wald Chi-square tests using *Anova* from the car package (16).

|  | **Substrate** | **Fixed Effect** | ***df*** | **Chisq** | ***p*-value** |
| --- | --- | --- | --- | --- | --- |
| **Species richness** | Xylose | Tillage | 1 | 13.374 | <0.001 |
|  |  | Day | 3 | 271.764 | <0.001 |
|  |  | Tillage:Day | 3 | 176.585 | <0.001 |
|  | Cellulose | Tillage | 1 | 5.3114 | 0.02 |
|  |  | Day | 3 | 150.9510 | <0.001 |
|  |  | Tillage:Day | 3 | 15.6767 | <0.001 |
| **Phylogenetic diversity** | Xylose | Tillage | 1 | 21.168 | <0.001 |
|  |  | Day | 3 | 671.424 | <0.001 |
|  |  | Tillage:Day | 3 | 405.437 | <0.001 |
|  | Cellulose | Tillage | 1 | 6.5313 | 0.01 |
|  |  | Day | 3 | 147.3154 | <0.001 |
|  |  | Tillage:Day | 3 | 37.7370 | <0.001 |

**Supplemental Figure 1. Total carbon (^12^C + ^13^C) mineralized (A) and mineralization rate (B) across tillage regimes and time since substrate addition.** Error bars represent standard deviation of mean rates and stars indicate significant post-hoc comparisons between tillage regimes derived from linear mixed effects models (Supplemental Table 2). Control microcosms did not receive carbon substrates and did not differ in mineralization rate between tillage regimes at any point during the incubation period.

**
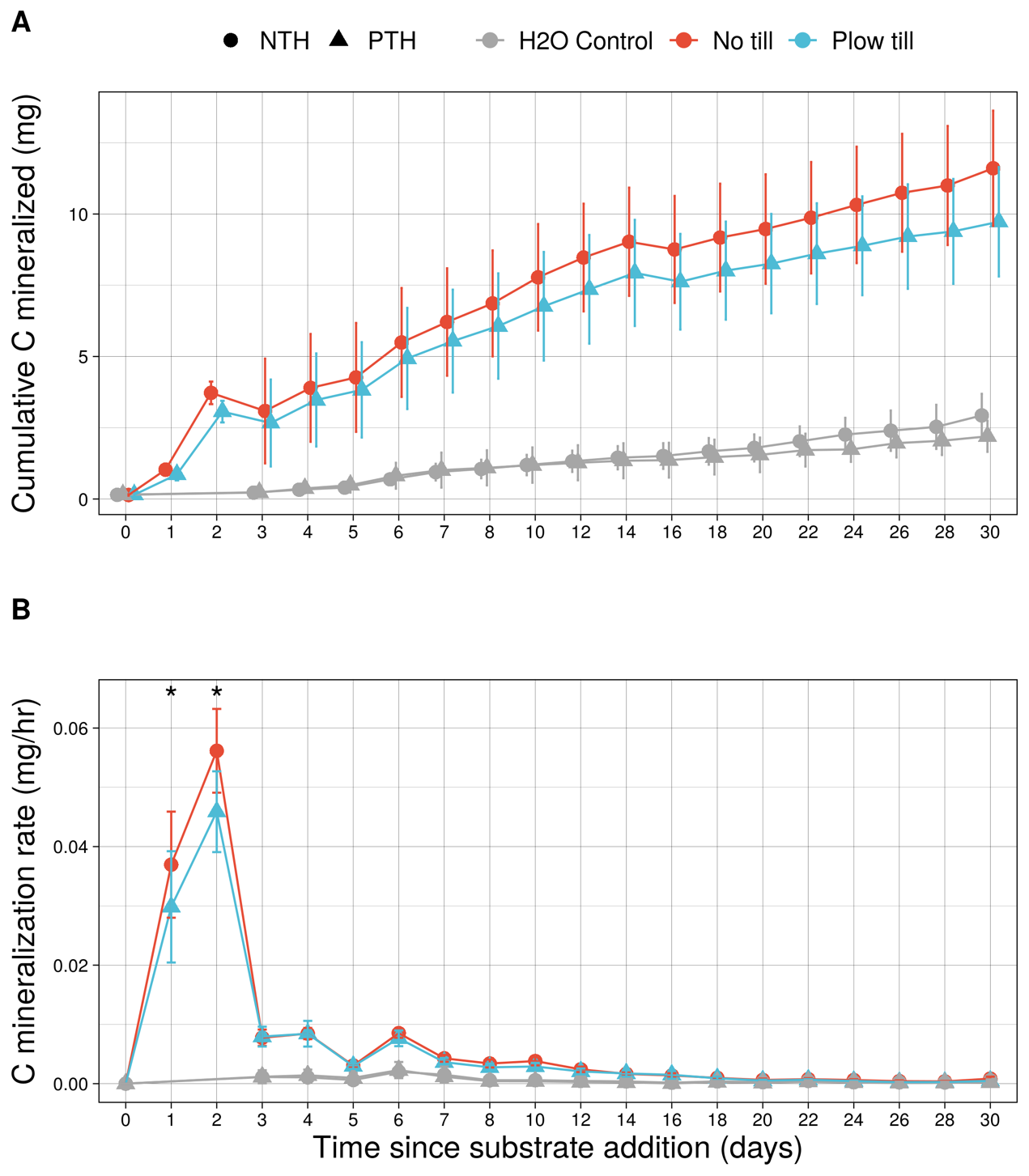
**

**Supplemental Figure 2. Cumulative mineralized carbon by individual substrate.** Total mineralized ^13^C-cellulose was determined by cumulative values up to sampling day 30, while mineralized ^13^C-xylose was determined by cumulative values up to day 14. Significant differences between tillage regimes were determined using posthoc means separation from linear mixed effect models (Supplemental Table 3) by sampling day (either 14 or 30) and substrate. Star indicates significantly different (*p < 0.05*) estimated marginal means at the end point, corrected for multiple comparisons.


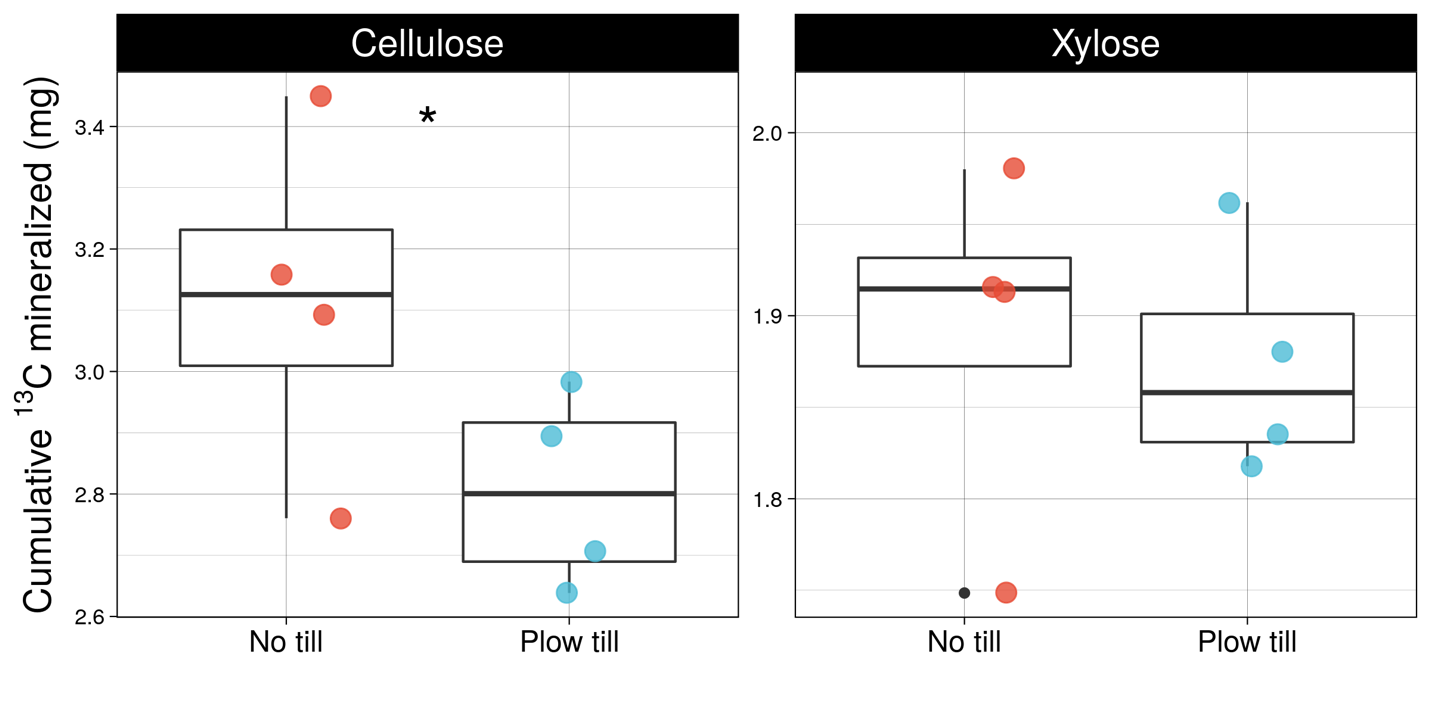


**Supplemental Figure 3. Differentially abundant taxa in divergent tillage regimes largely derived from the phylum *Proteobacteria*.** We identified differentially abundant taxa using a Maaslin2 (17) mixed model in which tillage, sampling day, and their interaction were specified as fixed effects. Microcosm replicate was specified as a random effect. The taxa are colored according to enrichment pattern. Taxa that were significantly enriched in one tillage regime (fixed effect = tillage) displayed significantly lower relative abundance in the divergent tillage regime. Differentially abundant taxa derived solely from the *Actinobacteriota*, *Bacteroidota*, *Firmicutes*, and *Proteobacteria* phlya.

**
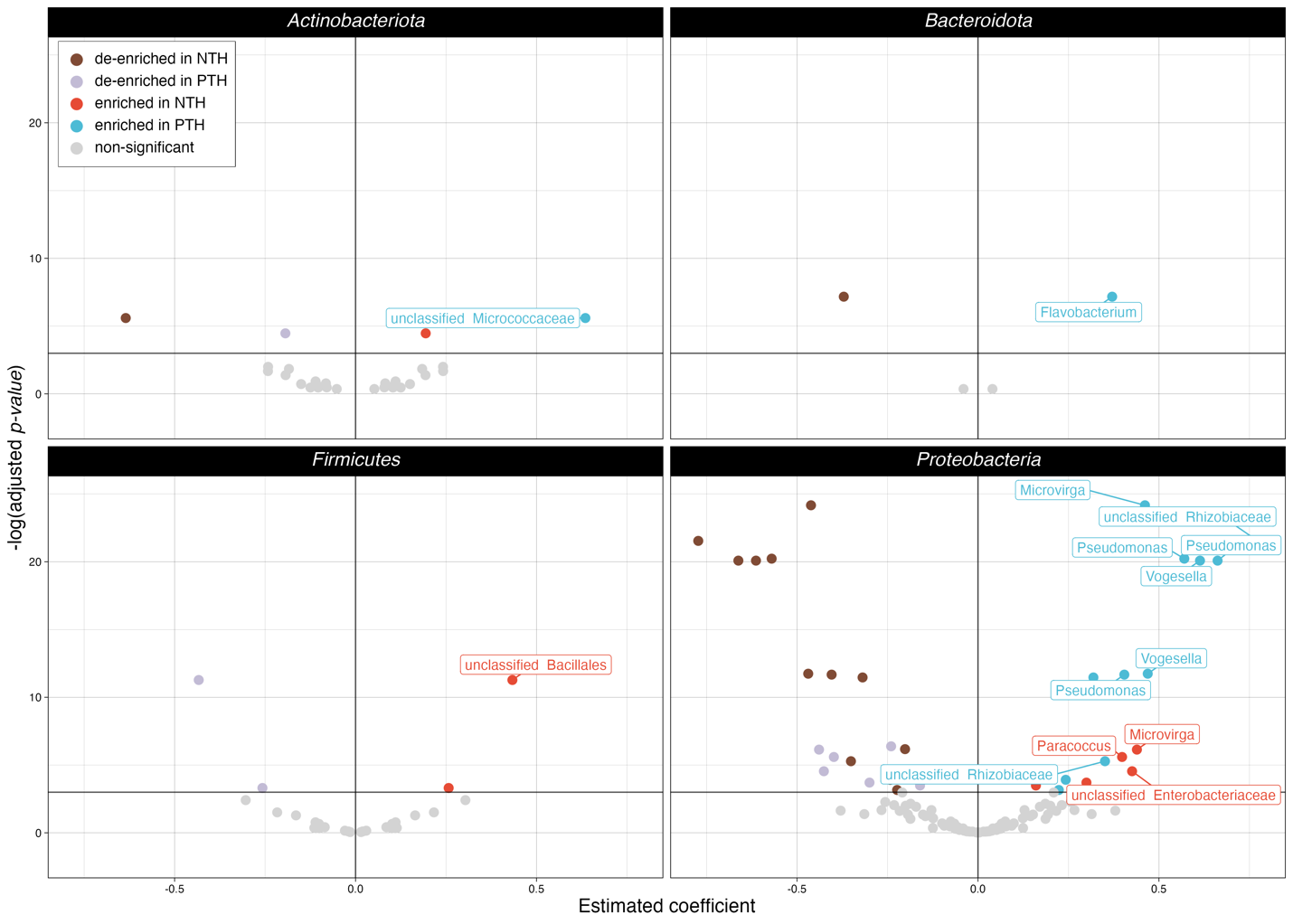
**

**Supplemental Figure 4. Carbon addition drives changes to microbial community structure irrespective of tillage regime.** Dashed lines represent the median distance among control microcosms that did not receive carbon inputs. Carbon addition resulted in rapid changes to community structure, with plotted data points indicating differences in beta diversity between microcosms receiving carbon and untreated controls receiving water. Significant differences in the distance between control and treated microcosms were determined via Wilcoxon rank-sum tests within tillage and sampling day. P-values were adjusted for multiple comparisons as described previously. Closed circles indicate significant differences in beta diversity compared to water-only controls.


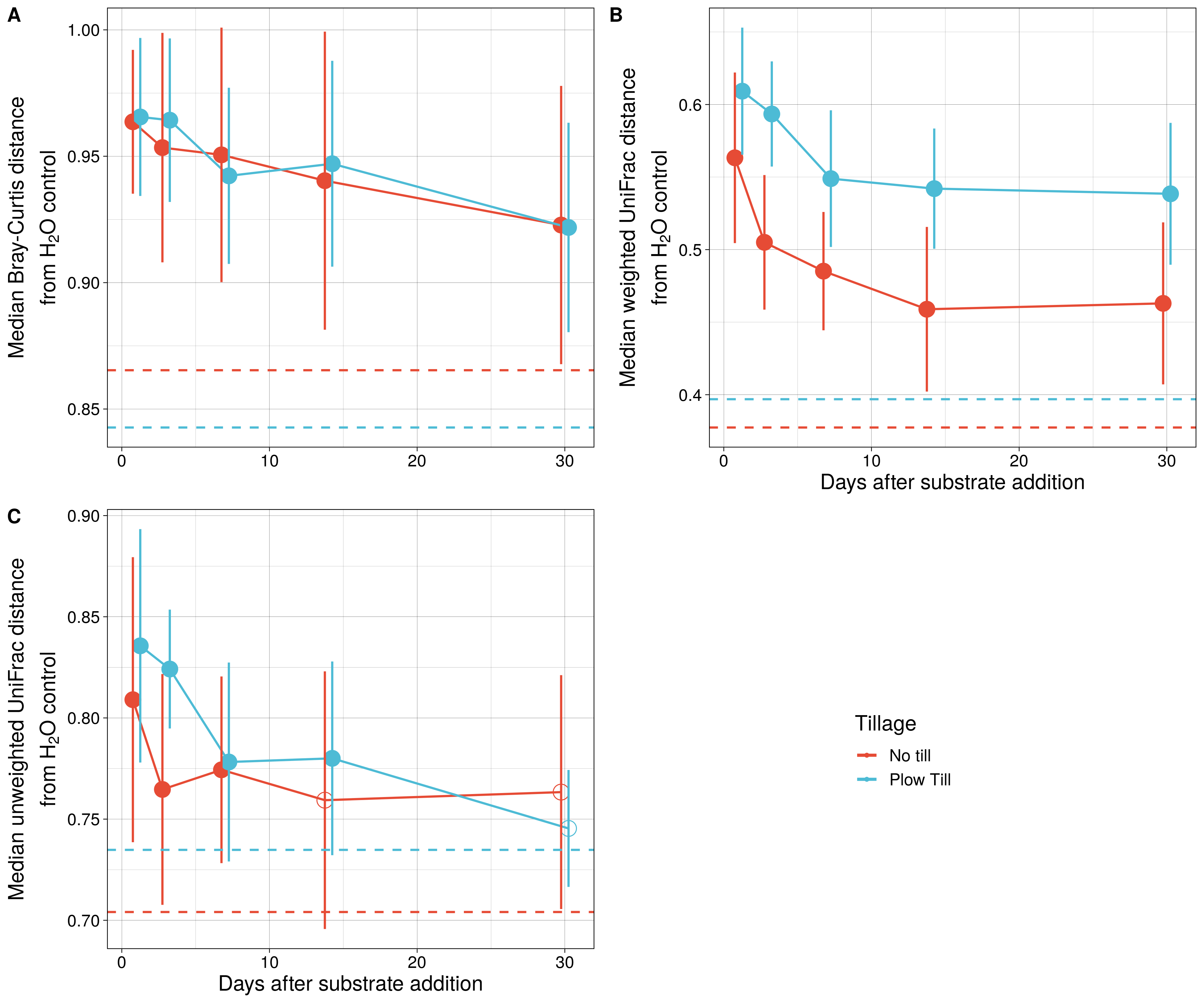


**Supplemental Figure 5. Incorporator diversity varies by labeled substrate, tillage regime, and days since C addition.** Significant differences in the phylogenetic diversity (A) and species richness (B) of incorporator taxa were determined by post-hoc tests of generalized linear mixed models (Supplemental Table 6).


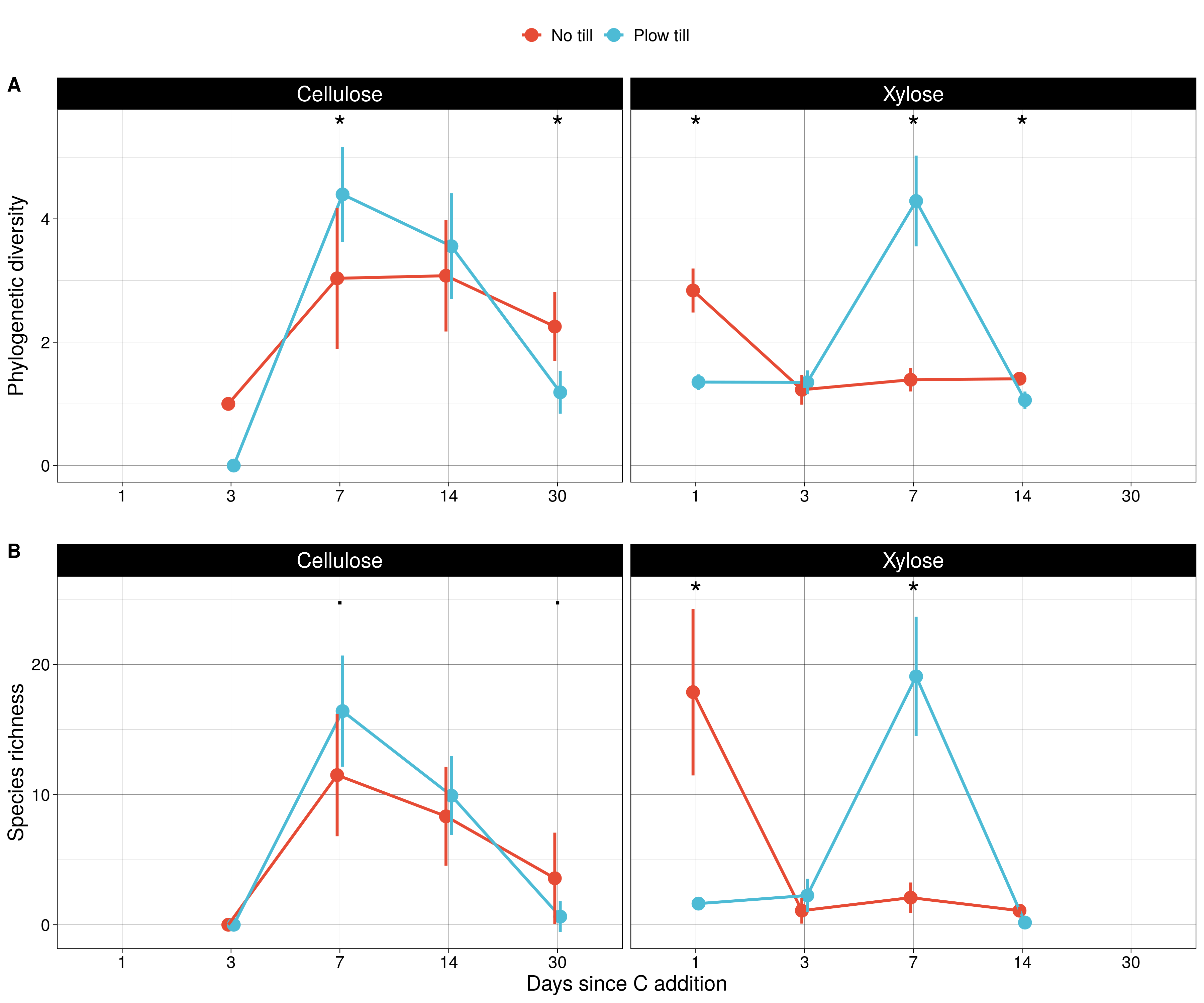


**Supplemental Figure 6. Predicted *rrn* differed reliably by carbon substrate across both tillage regimes.** Points and error bars denote the mean and standard deviation of predicted *rrn* between ^13^C labeled taxa within each tillage regime. In both tillage regimes, ^13^C-xylose assimilating taxa had significantly higher (*p* < 0.001) *rrn* based on Wilcoxon rank-sum tests.

**
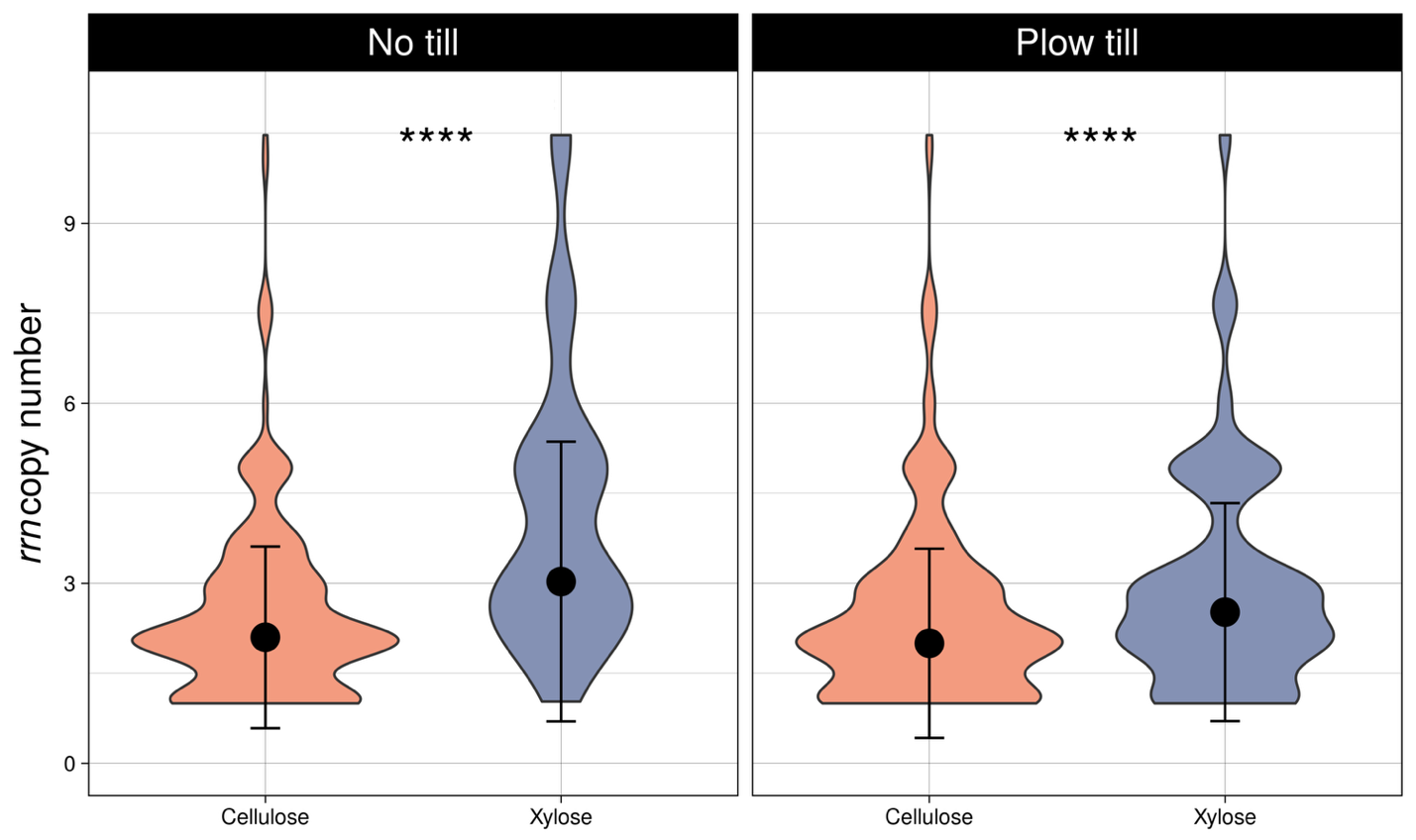
**

**Supplemental Figure 7. High *rrn* (> 3) and low *rrn* (< 3) incorporator taxa differ in their growth response by carbon substrate and tillage regime.** Normalized abundances were calculated as described previously by standardizing relative abundance with DNA yield and 16S rRNA copy number. The day of maximum observed abundance was determined for each incorporator ASV that was detected in rarefied microcosm soil communities. Differences in the growth by *rrn* category and tillage regime were probed with Fisher’s exact test (18).


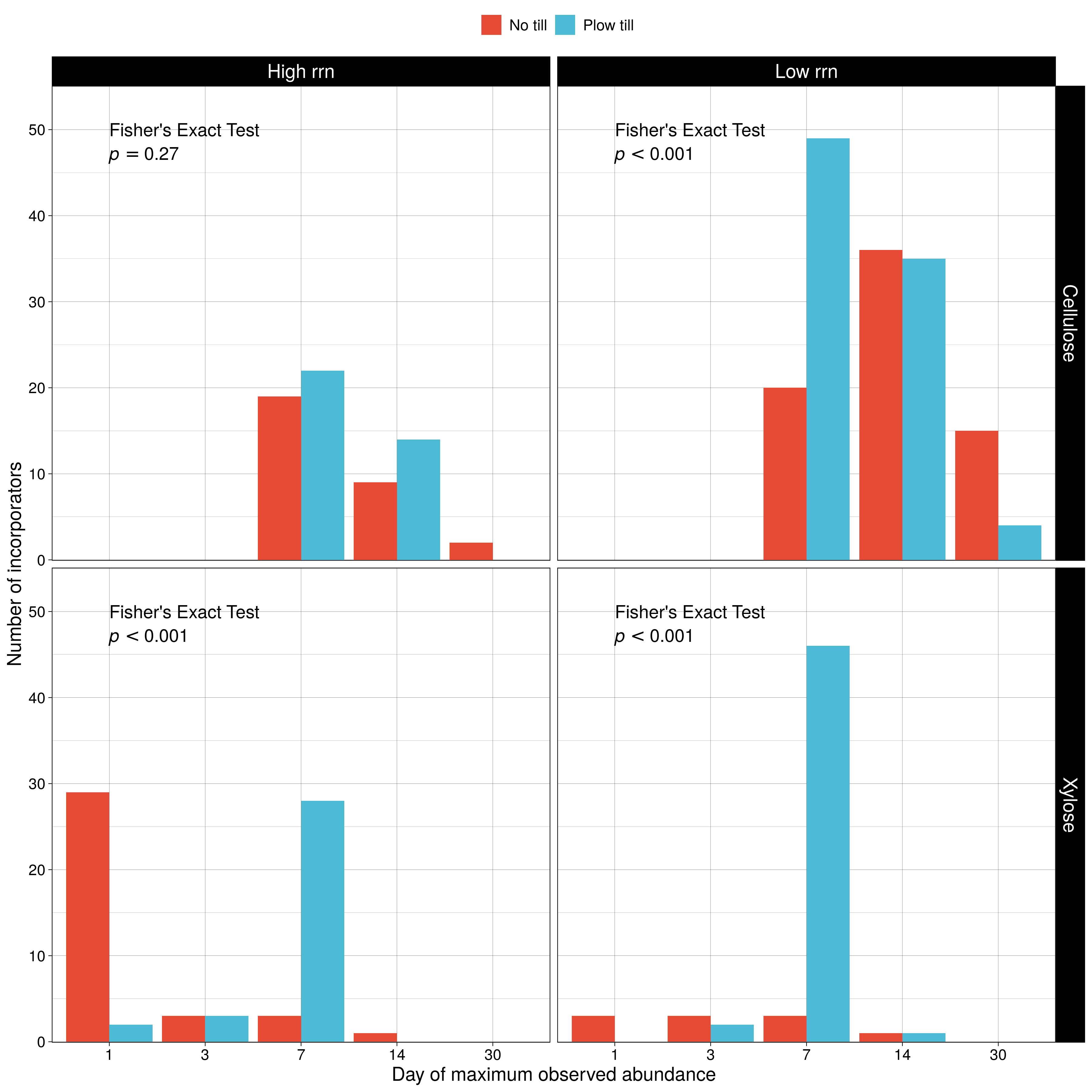


**Supplemental Figure 8. Functional characteristics related to carbon assimilation are poorly conserved among labeled taxa.** Functional distance was calculated based on incorporated substrate (^13^C-cellulose or ^13^C-xylose) day of labeling, and degree of ^13^C enrichment (see methods). Dashed lines denote expected distance based on average branch length for genus, family, order, class, and phylum (left to right). Incorporator ASVs beyond the genus level exhibit substantial functional dissimilarity.


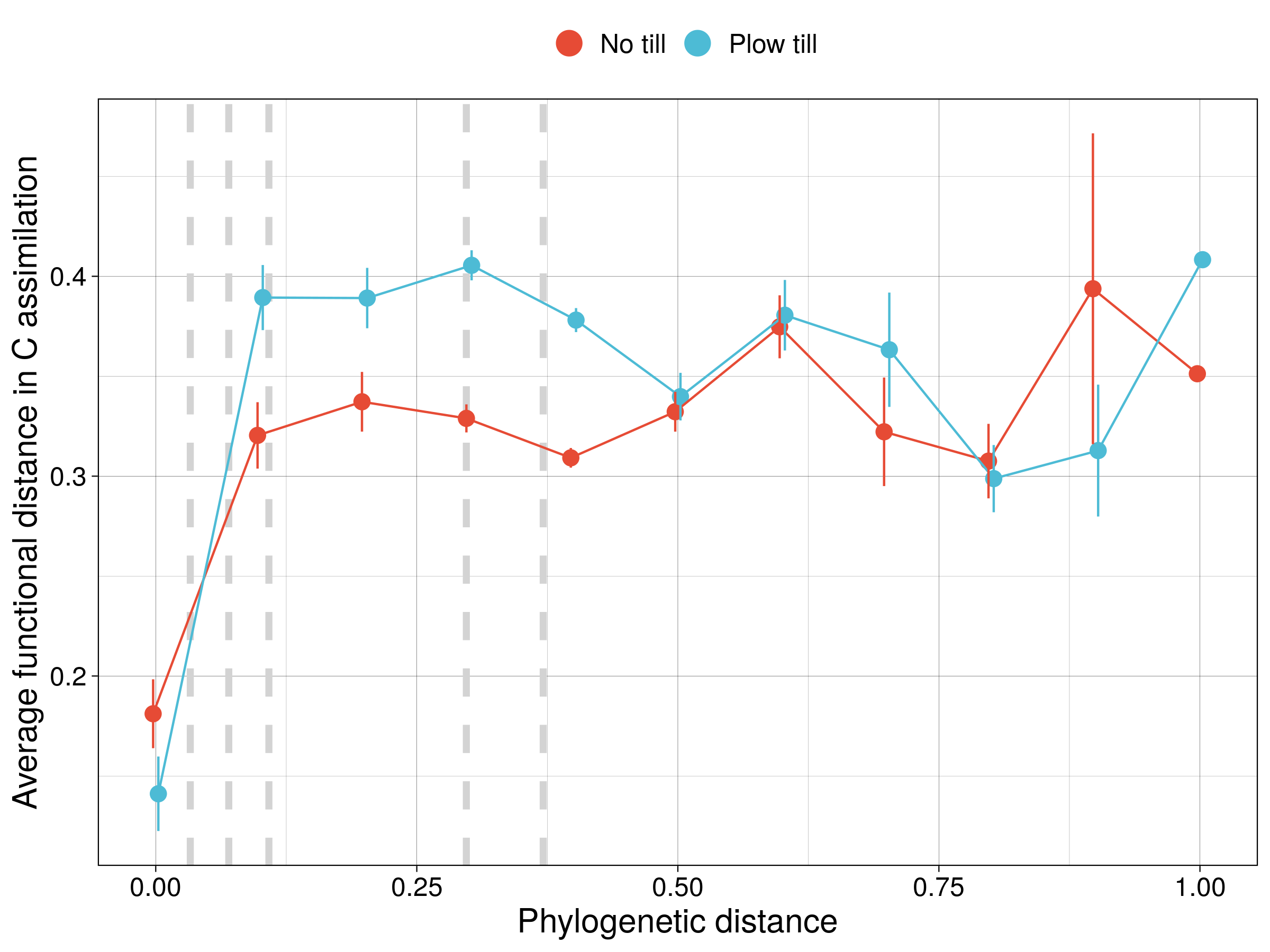


**Supplemental Figure 9. Log_2_-fold change of fungal OTU abundance in till (positive values) vs no till (negative values) arranged by fungal order for *Ascomycetes.*** Soil samples were collected from the long-term tillage experiment at Chazy, NY between July 2014 and November 2015. Internal transcribed spacer 1 (ITS1) amplicons were sequenced at the Cornell Core Facility in Ithaca, NY from extracted genomic soil DNA using the primer set nBITSf/58A2r (19, 20). Colors denote fungal order and significantly enriched OTUs are identified by an open dot. Figure obtained with permission from Koechli, 2016 (21).


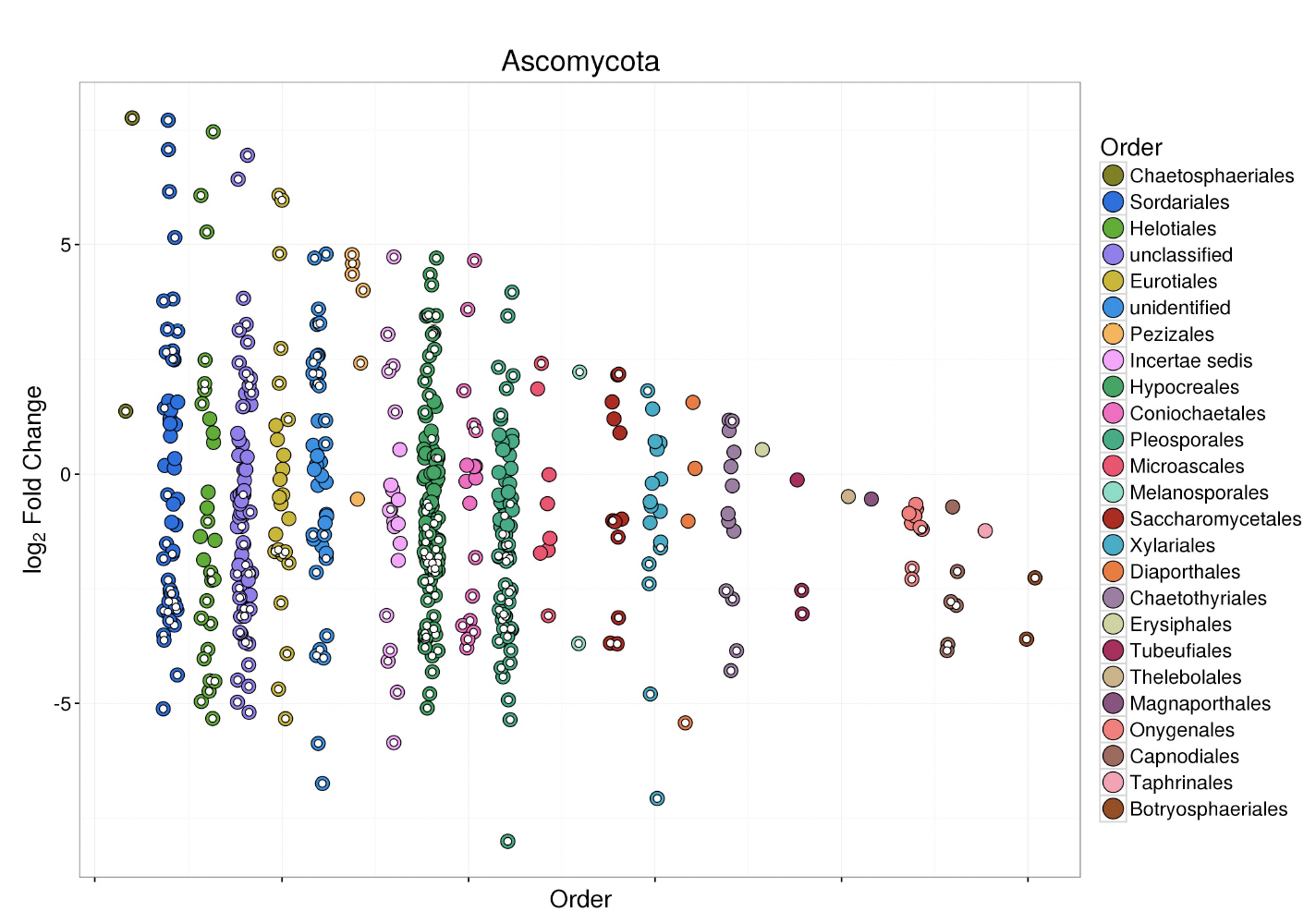


**Supplemental Figure 10. Log_2_-fold change of fungal OTU abundance in till (positive values) vs no till (negative values) arranged by fungal order for *Basidiomycetes.*** Soil samples were collected from the long-term tillage experiment at Chazy, NY between July 2014 and November 2015. ITS1 amplicons were sequenced at the Cornell Core Facility in Ithaca, NY from extracted genomic soil DNA using the primer set nBITSf/58A2r (19, 20). Colors denote fungal order and significantly enriched OTUs are identified by an open dot. Figure obtained with permission from Koechli, 2016 (21).


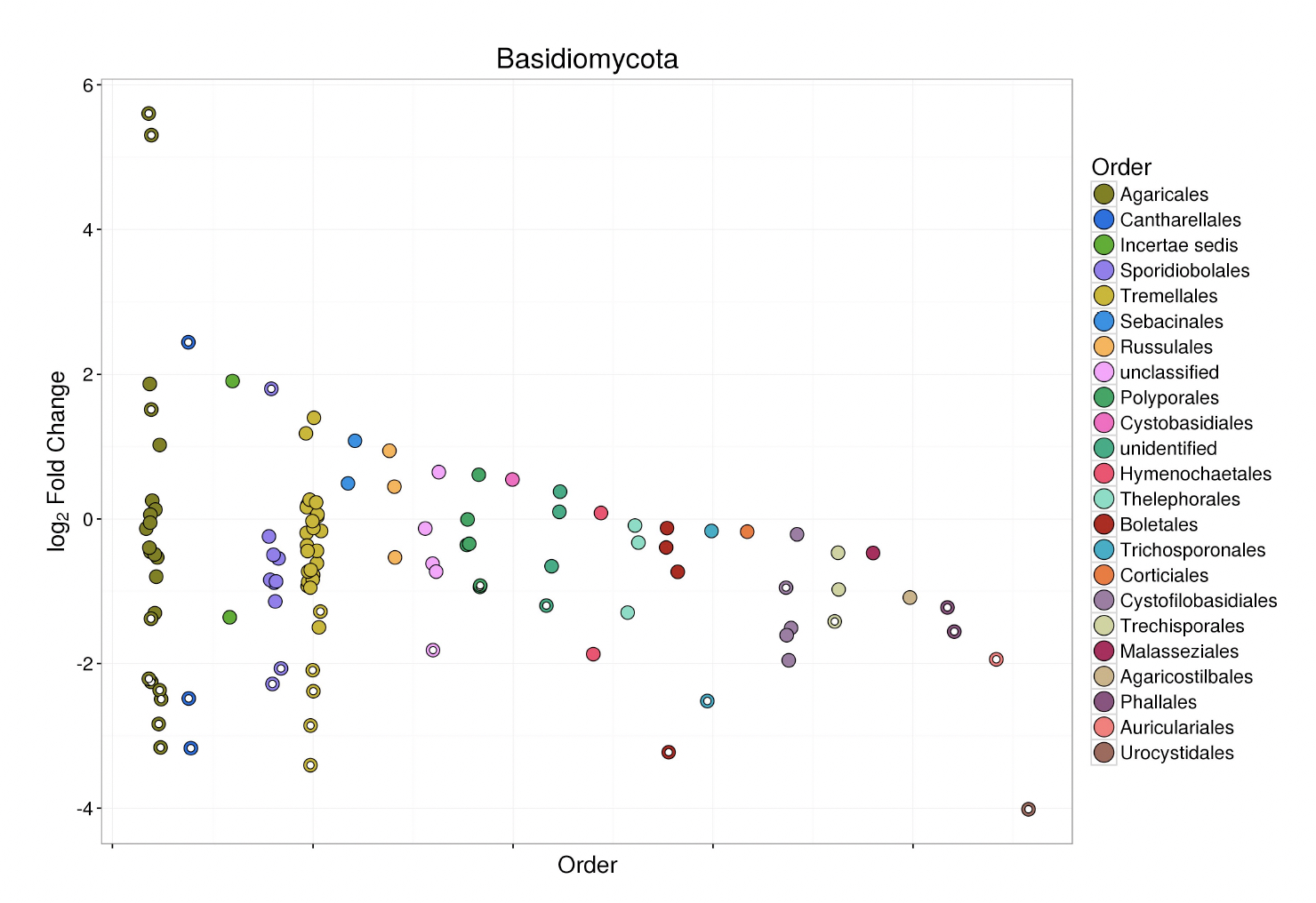


**References**

1. Berthrong ST, Buckley DH, Drinkwater LE. 2013. Agricultural Management and Labile Carbon Additions Affect Soil Microbial Community Structure and Interact with Carbon and Nitrogen Cycling. Microb Ecol 66:158–170.

2. Huang H-J, Ramaswamy S, Al-Dajani W, Tschirner U, Cairncross RA. 2009. Effect of biomass species and plant size on cellulosic ethanol: A comparative process and economic analysis. Biomass Bioenergy 33:234–246.

3. Pepe-Ranney C, Campbell AN, Koechli CN, Berthrong S, Buckley DH. 2016. Unearthing the Ecology of Soil Microorganisms Using a High Resolution DNA-SIP Approach to Explore Cellulose and Xylose Metabolism in Soil. Front Microbiol 7:703.

4. Barnett SE, Youngblut ND, Buckley DH. 2022. Bacterial community dynamics explain carbon mineralization and assimilation in soils of different land-use history. Environ Microbiol 24:5230–5247.

5. Barnett SE, Youngblut ND, Koechli CN, Buckley DH. 2021. Multisubstrate DNA stable isotope probing reveals guild structure of bacteria that mediate soil carbon cycling. Proc Natl Acad Sci 118:e2115292118.

6. Wilhelm RC, Barnett SE, Swenson TL, Youngblut ND, Koechli CN, Bowen BP, Northen TR, Buckley DH. 2022. Tracing Carbon Metabolism with Stable Isotope Metabolomics Reveals the Legacy of Diverse Carbon Sources in Soil. Appl Environ Microbiol 88:e00839-22.

7. Griffiths RI, Whiteley AS, O’Donnell AG, Bailey MJ. 2000. Rapid Method for Coextraction of DNA and RNA from Natural Environments for Analysis of Ribosomal DNA- and rRNA-Based Microbial Community Composition. Appl Environ Microbiol 66:5488–5491.

8. Neufeld JD, Dumont MG, Vohra J, Murrell JC. 2007. Methodological Considerations for the Use of Stable Isotope Probing in Microbial Ecology. Microb Ecol 53:435–442.

9. Manefield M, Whiteley AS, Griffiths RI, Bailey MJ. 2002. RNA Stable Isotope Probing, a Novel Means of Linking Microbial Community Function to Phylogeny. Appl Environ Microbiol 68:5367–5373.

10. Chaillou S, Pouwels PH, Postma PW. 1999. Transport of d-Xylose in Lactobacillus pentosus, Lactobacillus casei, andLactobacillus plantarum: Evidence for a Mechanism of Facilitated Diffusion via the Phosphoenolpyruvate:Mannose Phosphotransferase System. J Bacteriol 181:4768–4773.

11. Buckley DH, Huangyutitham V, Hsu S-F, Nelson TA. 2007. Stable Isotope Probing with 15N Achieved by Disentangling the Effects of Genome G+C Content and Isotope Enrichment on DNA Density. Appl Environ Microbiol 73:3189–3195.

12. Birnie GD. 1978. 6 - Isopycnic Centrifugation in Ionic Media, p. 167–217. *In* Centrifugal Separations in Molecular and Cell Biology. Butterworth-Heinemann.

13. Kozich JJ, Westcott SL, Baxter NT, Highlander SK, Schloss PD. 2013. Development of a dual-index sequencing strategy and curation pipeline for analyzing amplicon sequence data on the MiSeq Illumina sequencing platform. Appl Environ Microbiol 79:5112–5120.

14. Oksanen J, Simpson GL, Blanchet FG, Kindt R, Legendre P, Minchin PR, O’Hara RB, Solymos P, Stevens MHH, Szoecs E, Wagner H, Barbour M, Bedward M, Bolker B, Borcard D, Carvalho G, Chirico M, Caceres MD, Durand S, Evangelista HBA, FitzJohn R, Friendly M, Furneaux B, Hannigan G, Hill MO, Lahti L, McGlinn D, Ouellette M-H, Cunha ER, Smith T, Stier A, Braak CJFT, Weedon J. 2022. vegan: Community Ecology Package (2.6-2).

15. Bates D, Mächler M, Bolker B, Walker S. 2015. Fitting Linear Mixed-Effects Models Using lme4. J Stat Softw 67:1–48.

16. Fox J, Weisberg S. 2019. An R Companion to Applied RegressionThird. Sage, Thousand Oaks, CA. https://www.john-fox.ca/Companion/.

17. Mallick H, Rahnavard A, McIver LJ, Ma S, Zhang Y, Nguyen LH, Tickle TL, Weingart G, Ren B, Schwager EH, Chatterjee S, Thompson KN, Wilkinson JE, Subramanian A, Lu Y, Waldron L, Paulson JN, Franzosa EA, Bravo HC, Huttenhower C. 2021. Multivariable association discovery in population-scale meta-omics studies. PLoS Comput Biol 17:e1009442.

18. R Core Team. 2020. R: A language and environment for statistical computing. Vienna, Austria.

19. Bokulich NA, Mills DA. 2013. Improved Selection of Internal Transcribed Spacer-Specific Primers Enables Quantitative, Ultra-High-Throughput Profiling of Fungal Communities. Appl Environ Microbiol 79:2519–2526.

20. Martin KJ, Rygiewicz PT. 2005. Fungal-specific PCR primers developed for analysis of the ITS region of environmental DNA extracts. BMC Microbiol 5:28.

21. Koechli C. 2016. Land management afects microbial community composition and function in carbon cycling. Cornell University.
